# CD160-Derived Peptide as a Bidirectional Inhibitor Toward Immune Checkpoints BTLA/HVEM and HVEM/LIGHT

**DOI:** 10.1021/acs.jmedchem.5c02037

**Published:** 2025-11-30

**Authors:** Magdalena Lipińska, Piotr Ciura, Simon Gumpelmair, Emilia Sikorska, Katarzyna Kuncewicz, Adam K. Sieradzan, Peter Steinberger, Anna Wardowska, Marta Spodzieja

**Affiliations:** † Faculty of Chemistry, 49646 University of Gdańsk, Wita Stwosza 63, 80-308 Gdańsk, Poland; ‡ Institute of Immunology, Division of Immune Receptors and T Cell Activation, 27271 Medical University of Vienna, Lazarettgasse 19, 1090 Vienna, Austria; § Department of Rheumatology, Clinical Immunology, Geriatrics and Internal Medicine, 37804 Medical University of Gdańsk, Smoluchowskiego 17, 80-214 Gdańsk, Poland

## Abstract

BTLA and HVEM are key immune checkpoint proteins involved in regulating immune responses. Since HVEM also binds to CD160 at a site that overlaps with the BTLA binding site, CD160-derived fragments were used to design peptide inhibitors targeting this interface. Several peptides were synthesized and assessed for HVEM binding using SpS analysis, and their inhibitory activity was evaluated in ELISA and cell-based assays. One peptide, namely A5, demonstrated strong HVEM binding and effectively blocked the BTLA/HVEM interaction. Molecular docking results revealed that peptide A5 binds to HVEM not only at the BTLA interaction site but also at the region involved in LIGHT binding. Consistent with these findings, ELISA and cell-based assays confirmed that A5 effectively disrupts both BTLA/HVEM and HVEM/LIGHT complex formation. These results suggest that A5 acts as a dual inhibitor of HVEM interactions, with potential therapeutic implications for immune-related disorders.

## Graphical abstract

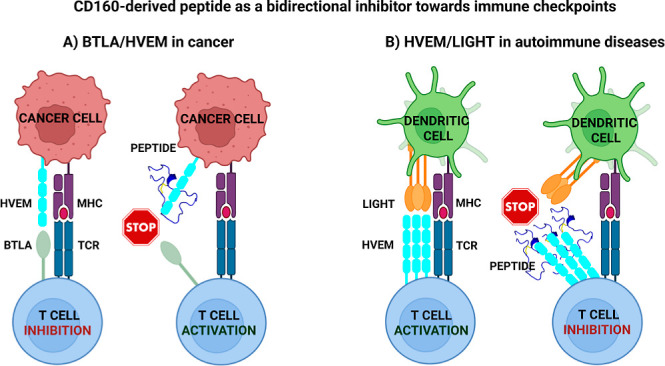

## Introduction

Immune checkpoints (ICPs) are essential for controlling the T cell-dependent immune response and maintaining the organism’s homeostasis. One can distinguish between costimulatory and coinhibitory ICPs. The former stimulate the activation of T cells, while the latter inhibit it.^1^ The complex formed by the proteins B- and T-lymphocyte attenuator (BTLA) and herpes virus entry mediator (HVEM) is among the inhibitory ICPs. The interaction of these proteins is crucial in regulating inflammatory, autoimmune, and antitumor responses, making the BTLA/HVEM complex a key focus of research and clinical investigations as a potential target for immunotherapy.^2–4^

BTLA is a receptor predominantly expressed on B and T cells, monocytes, macrophages, dendritic cells, and natural killer (NK) cells,^5^ while HVEM is found on the surface of T cells, B cells, and dendritic cells.^6^ Both are transmembrane proteins, and their extracellular domains mediate their interaction. The intracellular domain of BTLA contains two inhibitory motifs: an immunoreceptor tyrosine-based switch motif (ITSM) and an immunoreceptor tyrosine-based inhibitory motif (ITIM). The binding of the HVEM to the extracellular domain of the BTLA leads to the phosphorylation of tyrosine residues located within these motifs, which subsequently recruit protein tyrosine phosphatases possessing Src homology 2 (SH2) domains. These enzymes mediate immunosuppressive effects by dephosphorylating various proteins involved in intracellular signal transduction.^2^ Under physiological conditions, the interaction between BTLA and HVEM plays a crucial role in maintaining immune homeostasis and protecting the organism from autoimmunity. However, the upregulated expression of BTLA has also been reported on tumor-infiltrating lymphocytes (TILs), while HVEM is found on tumor cells. The binding of these proteins inhibits the activation, proliferation, and differentiation of T cells and is associated with an impaired antitumor immune response. This is considered one of the most important mechanisms by which tumor cells evade immune surveillance. Numerous reports confirm HVEM expression on tumor cells such as gastric cancer,^7,8^ colorectal cancer,^9^ esophageal squamous cell carcinoma,^10^ hepatocellular carcinoma,^11^ breast cancer,^12^ melanoma,^13^ ovarian cancer,^14^ and clear cell renal cell carcinoma.^15^ An increased level of BTLA on TILs has been observed in melanoma,^13^ nonsmall cell lung cancer,^16^ hepatocellular carcinoma,^17^ as well as on some cancer cells.^7,8^ The role of the BTLA/HVEM complex in autoimmune diseases remains to be fully elucidated. However, recent studies have shown that BTLA and HVEM can also be expressed on regulatory T cells (Tregs) in patients with systemic lupus erythematosus (SLE), and their interaction inhibits the function of these cells, thereby promoting disease progression.^18^ This suggests that blocking the BTLA/HVEM complex may have therapeutic potential not only in cancer immunotherapy but also in the treatment of autoimmune diseases; however, further research is needed.

BTLA belongs to the immunoglobulin superfamily (IgSF),^19^ while HVEM is a member of the tumor necrosis factor receptor superfamily (TNFRSF).^20^ In the extracellular part of HVEM, four cysteine-rich domains (CRDs) can be distinguished. The crystal structure of the BTLA/HVEM complex (PDB code: 2AW2) shows that a fragment 26–38, located in CRD1 of HVEM, interacts with two fragments in BTLA, corresponding to residues 35–43 and 118–128. These molecules form an antiparallel intermolecular β-sheet, which is stabilized mainly by interactions between the main chains of the proteins.^21^

Both proteins are part of a complex network and also interact with other molecules. BTLA binds to the UL144 protein from human cytomegalovirus (HCMV),^22,23^ while HVEM interacts with cluster of differentiation 160 (CD160),^24^ glycoprotein D (gD) from herpes simplex virus type 1/2 (HSV-1/2),^25^ LIGHT, and lymphotoxin α (LTα).^26^ The binding of HVEM to CD160 and BTLA inhibits T cell activation, whereas interaction with LIGHT and LTα promotes the activation of the immune system. Notably, different regions of HVEM are involved in binding to these molecules: BTLA, CD160, and gD primarily bind to the CRD1, while LIGHT and LTα mainly interact with CRD2 and CRD3 of HVEM.^27,28^

Our research focused on blocking the formation of the BTLA/HVEM complex using peptides. In order to achieve this, we used the amino acid sequence of the CD160 protein, whose binding site with HVEM overlaps with that of BTLA. CD160 is a member of the IgSF, and its structure resembles that of BTLA. CD160 interacts with CRD1 of HVEM, and the CD160/HVEM complex exhibits binding similarities to the BTLA/HVEM complex.^29^ The crystal structure of CD160/HVEM (PDB code: 6NG3) indicates that the following CD160 fragments are involved in interactions with CRD1 of HVEM: I27–S31 (A strand), R64–D67 (CC’ loop), Q111–R115 (F strand), S119–F127 (G^0^ and G strands), and K144 (H strand) in CD160. These regions form contacts with the following HVEM residues: K5, D7, E8, E14–P21, Y23, K26, E27, C29–L40.^29^

In the present study, based on the crystal structure of the CD160/HVEM protein complex and molecular mechanics-generalized Born surface area (MM-GBSA) analysis, we designed a series of peptides with the potential to inhibit BTLA/HVEM binding. These peptides were derived from two binding fragments of the CD160 protein and differed in amino acid sequence length and disulfide bond patterns. A comprehensive set of analyses was performed to evaluate their binding to the molecular target and their inhibitory properties. The results indicate that one of the designed peptides, referred to as A5, strongly interacts with HVEM and effectively blocks BTLA/HVEM complex formation in both enzyme-linked immunosorbent assays (ELISA) and cellular-based assays. Molecular dynamics (MD) simulations show that peptide A5 binds to the CRD1 of HVEM, overlapping with the BTLA binding site. However, A5 also engages the CRD2 and CRD3—regions involved in binding the LIGHT protein. This finding is consistent with our experimental results, which demonstrate that the peptide disrupts not only the BTLA/HVEM interaction but also the HVEM/LIGHT binding. Altogether, these data suggest that peptide A5 holds promise as a novel therapeutic agent for modulating immune responses. Further preclinical studies are required to fully evaluate its therapeutic potential.

## Results

### MM-GBSA Analysis for CD160/HVEM Complex

To enhance our understanding of the interaction between CD160 and HVEM, MM-GBSA analysis of the protein complex was performed, and two types of energy decomposition—per-residue and pairwise per-residue were calculated. The per-residue energy decomposition enables the determination of the contribution of individual amino acids in a protein complex by summing their interactions with all residues in the complex, whereas pairwise per-residue energy decomposition quantifies the interaction energy between individual pairs of amino acids within the complex.^30^ Per-residue analysis indicates that two regions in CD160 are involved in the interaction with HVEM. They are located in the middle part of the protein and at the C-terminus; however, the lowest energy values were assigned to amino acids located in the latter region (F, G^0^, and G strands) (Figure S1). Pairwise per-residue analysis strongly confirmed these results and additionally indicated that N28, located in the N-terminal fragment of the protein, plays a role in the CD160/HVEM complex formation (Figure S2).

The residues I121, R122, and Q124 in CD160 are crucial for binding to HVEM. Both I121 and R122 strongly interact with C37 of HVEM, while Q124 forms a contact with T35 in HVEM. Other important residues from the C-terminal part of CD160 include L123, H126, and F127. L123 binds to T35 in HVEM, whereas H126 and F127 interact with P17 in HVEM. Additionally, stabilization of the CD160/HVEM complex involves G120 and K144 from the C-terminal region, as well as S65 and G66 from the middle part of the CD160 protein. G120 interacts with Y23 in HVEM, K144 binds to E31 in HVEM, and S65 and G66 form contacts with D7 and S20 in HVEM. Amino acids from the N-terminal part of CD160 appear to be less critical for protein binding than those described above. A significant interaction contributing to protein complex stabilization was observed only for N28, which interacts with V36 (Table S1). The most important amino acids, identified through per-residue and pairwise per-residue energy decomposition, are presented in Table 1. Additionally, energy decomposition divided into van der Waals + nonpolar and electrostatic + polar components^31,32^ and obtained from the MM-GBSA analysis, have also been included in the Supporting Information (Table S2, Figures S3 and S4). The results indicate that van der Waals and nonpolar interactions play a key role in stabilizing the protein/protein complex, highlighting the importance of hydrophobic residues for binding, although a few strong electrostatic interaction pairs are also present.

**Table 1:** The Amino Acid Residues Crucial for CD160/HVEM Complex Formation Identified Through the MM-GBSA Energy Decomposition Analysis^33^ ^,^ ^a^

analysis	crucial CD160 residues involved in CD160/HVEM complex formation	crucial HVEM residues involved in CD160/HVEM complex formation
Per-residue	**S65**, **G66**, **G120**, **I121**, **R122**, **L123**, **Q124**, **H126**, **F127**, **K144**	**D7**, **P17**, **Y23**, **L32**, **T33**, **G34**, **T35**, **V36**, **C37**, P39, L49
Pairwise per-residue	N28, **S65**, **G66**, **G120**, **I121**, **R122**, **L123**, **Q124**, **H126**, **F127**, **K144**	**D7**, **P17**, S20, **Y23**, E31, **L32**, **T33**, **G34**, **T35**, **V36**, **C37**, E38

a Residues with the strongest influence on complex formation in both decomposition methods are highlighted in bold. The criterion for “strong” interaction energy was defined as an energy of −1 kcal/mol or lower for per-residue decomposition, and −3 kcal/mol or lower for pairwise per-residue decomposition. The same criteria were used as in our previous study describing the BTLA/HVEM complex.

### Design of Potential CD160-Derived Peptide Inhibitors of BTLA/HVEM Complex Formation

The region of HVEM recognized by CD160 overlaps with the BTLA binding site. Therefore, to design peptides that act as potential inhibitors of BTLA/HVEM complex formation, CD160 fragments that interact with HVEM were used. Based on the crystal structure of CD160/HVEM (PDB code: 6NG3) and MM-GBSA analysis performed for this complex, three groups of peptides (A, B, and C) were designed (Figure 1), with their amino acid sequences listed in Table 2. Group A includes peptides derived from the C-terminal part of CD160, covering residues 110–128, while groups B and C contain the peptides based on the middle part of CD160. Group B covers amino acids 56–79, whereas group C peptides are shortened versions of group B and include residues 59–75. The N-terminal fragment of CD160 was not used for peptide design, as both per-residue and pairwise per-residue energy analyses indicated that residues in this region interact with HVEM but are not essential for protein complex formation. The amino acid sequence of native CD160 contains five cysteine residues, which form two disulfide bonds: C44–C112 and C61–C68, while the cysteine at position 113 remains unpaired.^24,34^ In the designed peptides, these cysteines were either used to form disulfide bonds or substituted by 2-aminobutanoic acid (X). In most of the designed peptides, disulfide bonds were introduced by substituting selected residues with cysteines. This modification aimed to stabilize the β-hairpin structure formed by the corresponding fragment in native CD160. Cysteine substitutions were introduced at positions where the side chains of selected amino acids are opposite each other in the crystal structure, enabling disulfide bond formation with minimal impact on peptide conformation. However, in some cases, introducing disulfide bonds required the substitution of residues critical for interaction with HVEM.

**Figure 1:**
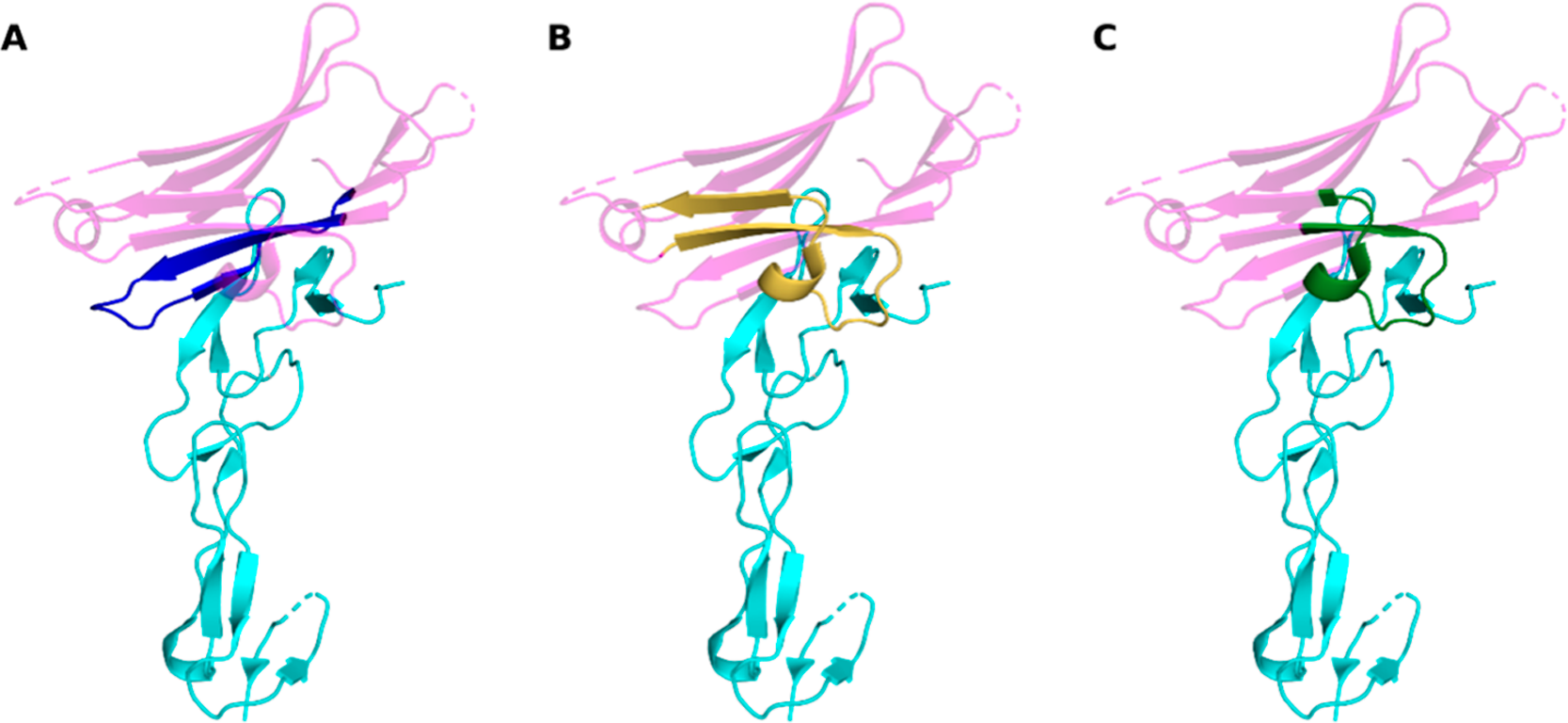
Structure of the CD160/HVEM complex (PDB code: 6NG3). CD160 is shown in magenta and HVEM in cyan. The CD160 fragments used for peptide design are highlighted: (A) residues 110–128 (group A, dark blue), (B) residues 56–79 (group B, yellow), and (C) residues 59–75 (group C, dark green).

**Table 2:** Amino Acid Sequences of the Designed CD160-Derived Peptides, X—2-Aminobutanoic Acid, &—Position of Disulfide Bond

no.	abbreviation	peptide name	amino acid sequence
1	A1	CD160(110–128)^(C112X,C113X)^	Ac-YQXXARSQKSGIRLQGHFF-NH_2_
2	A2	CD160(110–128)^(Q111C,C112X,C113X,F127C)^	Ac-YC(&)XXARSQKSGIRLQGHC(&)F-NH_2_
3	A3	CD160(110–128)^(C112X,Q124C)^	Ac-YQXC(&)ARSQKSGIRLC(&)GHFF-NH_2_
4	A4	CD160(110–128)^(C112X,C113X,A114C,L123C)^	Ac-YQXXC(&)RSQKSGIRC(&)QGHFF-NH_2_
5	A5	CD160(110–128) ^(C113X,G125C)^	Ac-YQC(&)XARSQKSGIRLQC(&)HFF-NH_2_
6	B1	CD160(56–79)^(C61X,C68X)^	Ac-FVVFLXKDRSGDXSPETSLKQLRL-NH_2_
7	B2	CD160(56–79)^(V57C,C61X,C68X,R78C)^	Ac-FC(&)VFLXKDRSGDXSPETSLKQLC(&)L-NH_2_
8	B3	CD160(56–79) ^(L60C,C61X,C68X,L74C)^	Ac-FVVFC(&)XKDRSGDXSPETSC(&)KQLRL-NH_2_
9	B4	CD160(56–79)^(F59C,C61X,C68X,Q76C)^	Ac-FVVC(&)LXKDRSGDXSPETSLKC(&)LRL-NH_2_
10	B5	CD160(56–79)	Ac-FVVFLC(&)KDRSGDC(&)SPETSLKQLRL-NH_2_
11	C1	CD160(59–75)^(C61X,C68X)^	Ac-FLXKDRSGDXSPETSLK-NH_2_
12	C2	CD160(59–75)^(L60C,C61X,L74C)^	Ac-FC(&)XKDRSGDXSPETSC(&)K-NH_2_
13	C3	CD160(59–75)	Ac-FLC(&)KDRSGDC(&)SPETSLK-NH_2_

### Binding Affinities of CD160-Derived Peptides to HVEM Determined by SpS

In the initial phase of studies, the binding of the designed peptides to HVEM was evaluated using the spectral shift (SpS) technique. HVEM protein with a His tag was labeled with a RED-tris-NTA second Generation dye and incubated with the peptides at 16 different concentrations. The samples were transferred into capillaries, and the ratio of fluorescence intensity at two emission wavelengths was measured as a function of the peptide concentration. The resulting spectral shift dose–response curves indicated concentration-dependent binding of peptides A4 (Figure 2A) and A5 (Figure 2B) to the target protein. Peptide A5 exhibited the strongest affinity to HVEM, with a dissociation constant (K_d_) of 1.38 μM. Peptide A4 showed the K_d_ of 231 μM; however, under the tested conditions, a plateau was not reached. Therefore, the reported value for peptide A4 represents an apparent rather than a true dissociation constant. The remaining peptides did not show detectable binding to HVEM under the experimental conditions. For comparison, a similar assay was performed to assess the interaction between BTLA and HVEM, and the determined K_d_ was 0.31 μM (Figure 2C). The K_d_ values were converted into binding free energies (ΔG) and compared with those obtained from MM-GBSA analysis (Table S3), and discussed in detail in Supporting Information.

**Figure 2:**
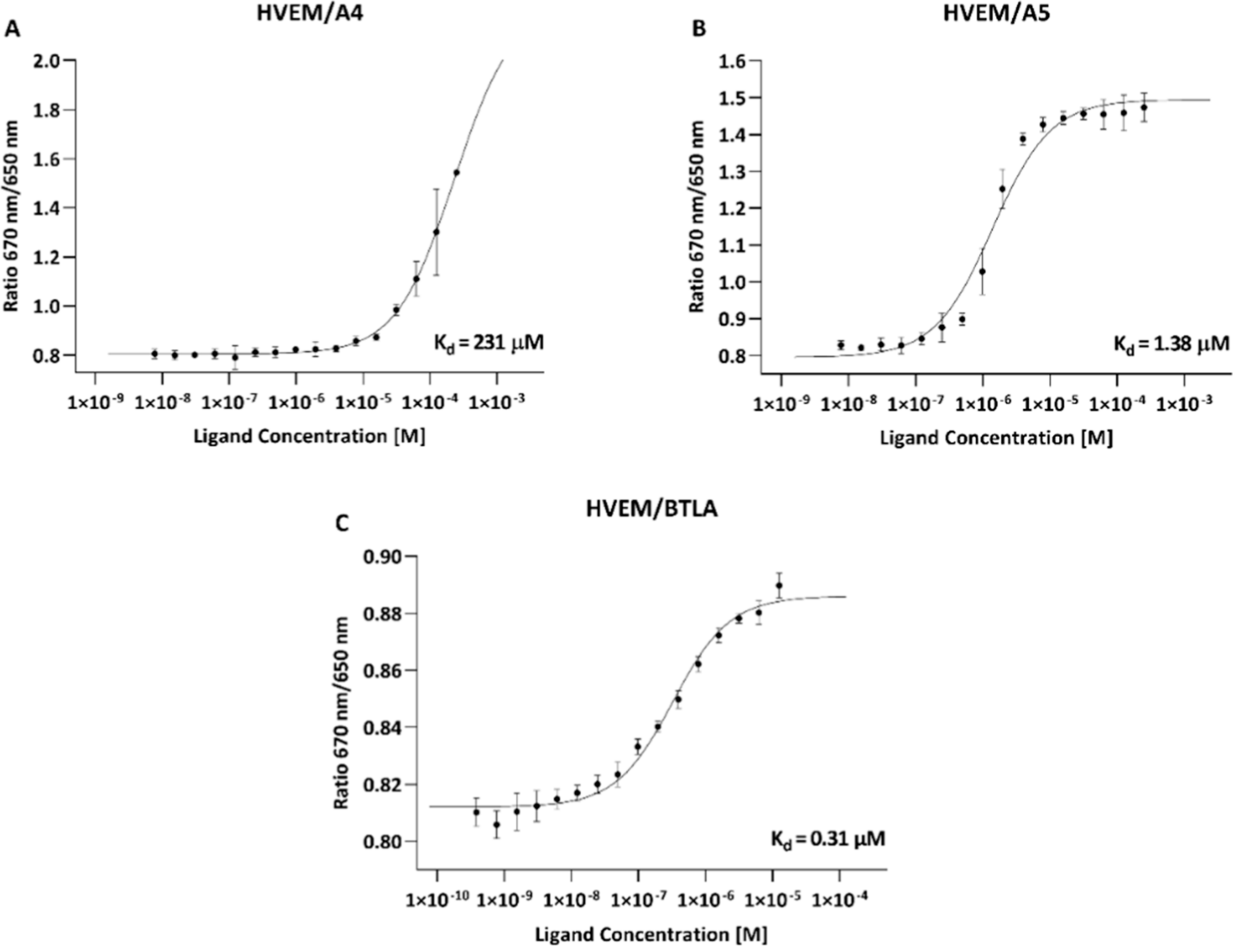
Spectral shift dose–response curves showing the interactions between HVEM and: (A) peptide A4, K_d_ = 231 μM, 95% confidence interval (CI): 146–365 μM; (B) peptide A5, K_d_ = 1.38 μM, 95% CI: 1.09–1.74 μM; (C) BTLA, K_d_ = 0.31 μM, 95% CI: 0.25–0.39 μM. The confidence interval was calculated from the variance of the fitted parameter, as determined using the Levenberg–Marquardt algorithm in the MO.Control v2.5.4 software.

### Inhibitory Properties of the CD160-Derived Peptides toward BTLA/HVEM Complex Determined in ELISA

In the subsequent stage of the study, the potential of the obtained compounds to inhibit the interaction between BTLA and HVEM proteins was evaluated using ELISA, following the methodology described in our previous studies.^33,35,36^ HVEM was immobilized on the plate and incubated with peptides at three different concentrations, followed by the addition of Fc-tagged BTLA. Detection was carried out using horseradish peroxidase (HRP) conjugated with anti-human IgG and 3,3′,5,5′-tetramethylbenzidine (TMB) as a substrate. Signal intensity was directly proportional to the amount of BTLA bound to HVEM and inversely proportional to the inhibitory activity of the tested peptides. As a control, BTLA and HVEM were incubated in the absence of peptides.

The results indicate that peptide A5 inhibits BTLA/HVEM protein binding in a concentration-dependent manner. At 150 μM, it achieved approximately 98% inhibition, while at 50 μM, the inhibition was around 36%. Peptides B1 and B2 also showed inhibitory activity at the highest tested concentration, reducing the binding of BTLA to HVEM by approximately 24% and 14%, respectively. The remaining compounds exhibited negligible or no detectable inhibitory effects under the tested conditions (Figure 3).

**Figure 3:**
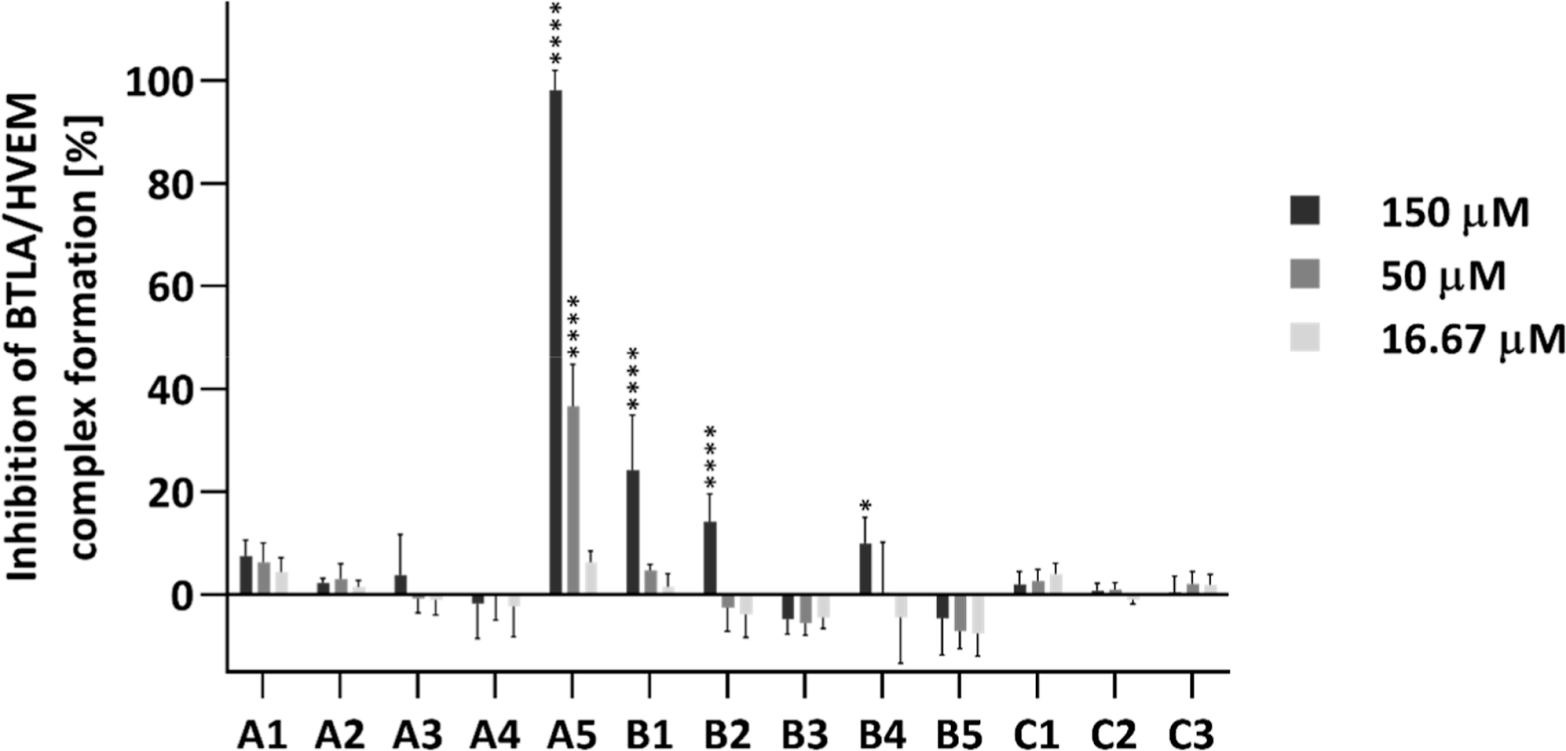
The inhibitory properties of the CD160-derived peptides toward the BTLA/HVEM complex formation determined using ELISA. Results are shown for three experiments performed independently. BTLA/HVEM binding in the absence of peptides was used as the reference and defined as 0% inhibition. Data are depicted as mean with standard deviation (SD). Statistical analysis was performed using one-way analysis of variance (ANOVA) followed by Dunnett’s post hoc test. ****p < 0.0001, ***p < 0.001, **p < 0.01, *p < 0.05.

### Inhibitory Properties of the CD160-Derived Peptides toward BTLA/HVEM Determined in Cellular Assays

Before conducting cellular assays, the effects of the peptides on cell viability in Jurkat E6.1 and T cell stimulator (TCS) cell lines, as well as their stability in cell culture supernatant, were evaluated. Jurkat E6.1 cells are human T cell acute lymphoblastic leukemia cells, while TCS is a modified murine thymoma cell line (BW5147). The cellular platforms described below are based on these cells. Most peptides did not significantly affect cell viability, except peptide B2, which markedly reduced the viability of Jurkat E6.1 and TCS cells to 47% (Figure S5A) and 9% (Figure S5B), respectively, at the highest tested concentration (150 μM). Due to its cytotoxicity, this concentration of B2 was excluded from further experiments. Peptide B5 also exhibited some cytotoxicity toward Jurkat E6.1 cells at the highest concentration; however, cell viability remained relatively high at 75% after 24 h of incubation (Figure S5A). The stability of the peptides in supernatant collected from cells after 24 h of incubation was also assessed. Most peptides were stable under these conditions but showed binding to components of the culture medium. This was reflected by a decrease in peak area on chromatograms, without the appearance of additional signals indicative of peptide degradation (Figure S6). A representative chromatogram of peptide A5, which demonstrated the strongest binding to HVEM in SpS and the most potent inhibitory activity in ELISA, is shown in Figure S7.

In the next step of the study, the inhibitory properties of the designed peptides were evaluated using previously described cell-based assays.^37^ The experiment utilized reporter cells expressing HVEM (JE6.1-NF-κB::eGFP) and T cell stimulator cells expressing BTLA (TCS-BTLA). Activation of the reporter cells by TCS followed a two-signal model. The first is generated through the interaction between the T cell receptor–cluster of differentiation 3 (TCR–CD3) complex on the surface of the reporter cells and a membrane-bound antihuman CD3 single-chain antibody fragment (mAb aCD3) expressed on the TCS cells. The second signal is provided by the binding of HVEM and BTLA, which in this assay results in a strong costimulatory signal via HVEM. Activation of the reporter cells leads to the expression of nuclear factor kappa light chain enhancer of activated B cells (NF-κB), which correlates with the level of enhanced green fluorescent protein (eGFP), measured by flow cytometry. In the absence of peptides, NF-κB expression is normalized to 100%. Disruption of the BTLA/HVEM interaction by peptides results in a concentration-dependent decrease in NF-κB levels. In this study, HVEM-expressing reporter cells were stimulated with BTLA-expressing TCS cells in the presence or absence of tested peptides at three different concentrations.

The results showed that peptide A5 strongly inhibits the BTLA/HVEM interaction, reducing NF-κB activation in a dose-dependent manner: approximately 81% inhibition at 150 μM, 60% at 50 μM, and 14% at 16.67 μM. Peptide B1 demonstrated slight inhibitory activity, only at the highest concentration tested. The remaining peptides exhibited either negligible or no inhibitory effects (Figure 4). The highest concentration of peptide B2 was excluded from testing due to its cytotoxicity in both cell lines.

**Figure 4:**
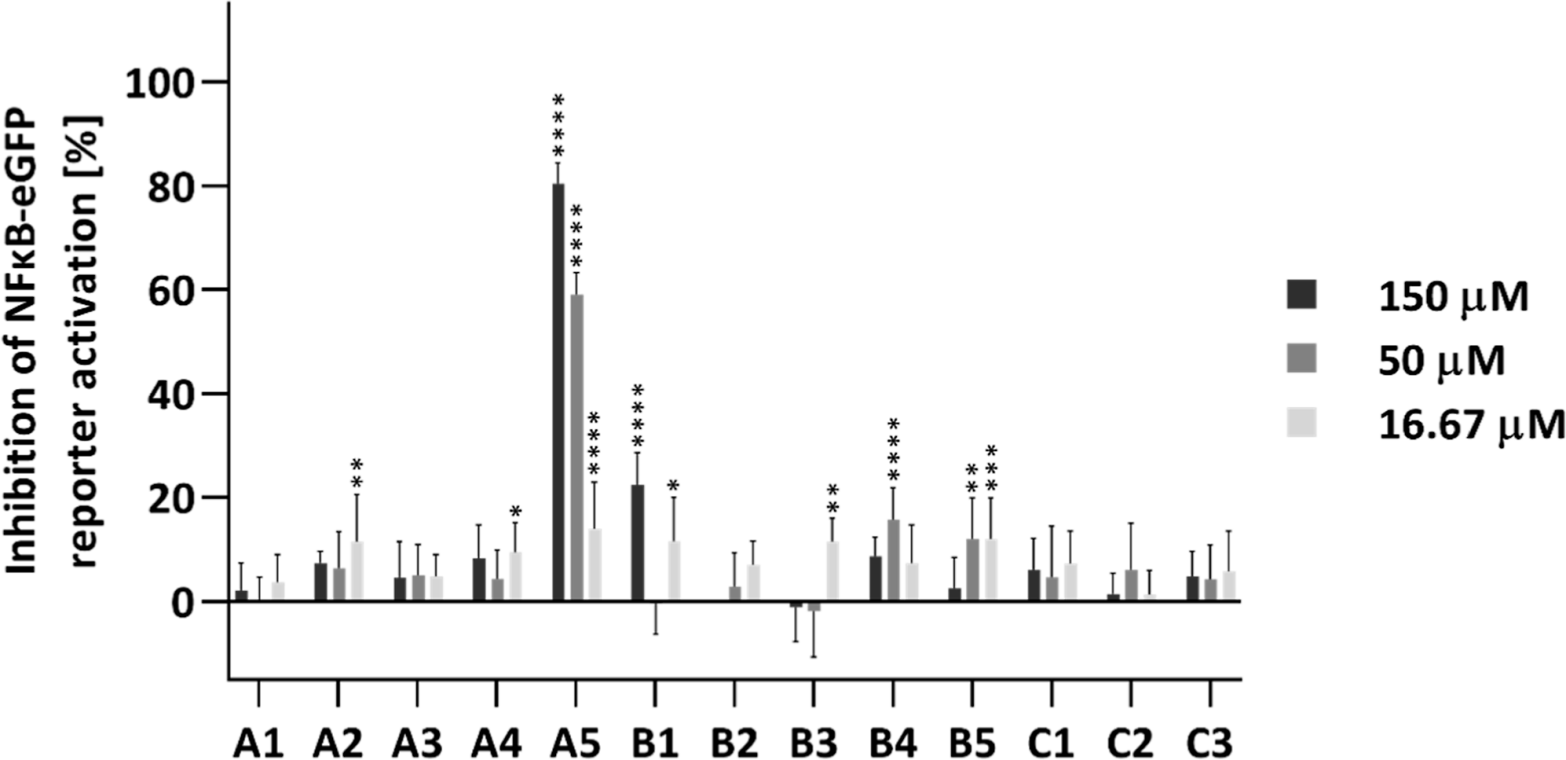
Inhibitory properties of the CD160-derived peptides toward the BTLA/HVEM complex formation measured in cellular-based assays. Results are shown for four experiments performed independently in duplicate. Data are depicted as mean with SD. Statistical analysis was performed using one-way ANOVA followed by Dunnett’s post hoc test. ****p < 0.0001, ***p < 0.001, **p < 0.01, *p < 0.05.

### Conformational Studies of Peptide A5—the Best Inhibitor of the BTLA/HVEM Complex Formation Using CD, NMR and Molecular Dynamics

Since peptide A5 was identified as the most effective inhibitor of BTLA/HVEM binding, a detailed conformational analysis was subsequently performed. First, circular dichroism (CD) spectra were recorded for this peptide in H_2_O and phosphate-buffered saline (PBS). In water, the peptide adopts a disordered structure, characterized by a minimum at 197 nm. In contrast, in PBS, it undergoes conformational ordering, forming a β-strand structure with a minimum at 214 nm and maximum at 197 nm. A detailed analysis of the conformational changes observed for the peptide A5 in H_2_O and in PBS is provided in the Supporting Information (Figure S8), revealing β-sheet promoting properties of phosphate ions. To further investigate the peptide’s structure, nuclear magnetic resonance (NMR) spectra were acquired in both buffer-free aqueous solution and PBS-buffered aqueous solution. In PBS, extensive signal broadening indicated peptide aggregation; therefore, structural analysis was conducted based on the spectra recorded in the buffer-free aqueous solution. The assignment of proton resonances for A5 peptide was carried out following standard protocols, utilizing 2D total correlation spectroscopy (TOCSY) and nuclear Overhauser effect spectroscopy (NOESY) data sets (Table S4), and further supported by a heteronuclear 2D ^1^H–^13^C heteronuclear single quantum correlation (HSQC) experiment. Narrow amide proton chemical shift dispersion (Δδ_HN_ = 0.7 ppm), absence of medium- and long-range nuclear Overhauser effects (NOEs), and ^3^J_Nα_ coupling constants indicate a lack of well-defined secondary structure (Figures S9 and S10). However, local HN–HN (i, i + 1) NOE connectivities in the 10–16 fragment (S120–C126 in CD160) suggest turn-like motifs. Molecular dynamics simulations with NOE-derived distance restraints generated an ensemble of structures showing high conformational diversity. From 1000 generated structures, 133 low-energy models were selected based on a total distance penalty not exceeding 0.5 kcal/mol and were grouped into 10 families, with one dominant cluster containing 67% of the structures (Table S5). The root-mean-square deviation (RMSD) for backbone atoms in the cyclic C3–C16 part within the dominant family is 2.329 ± 0.329 Å, indicating significant structural variability. The Ramachandran plot and an average radius of gyration (R_g_ = 9.3 ± 0.5 Å) confirm the dynamics and flexibility of the peptide (Figure S11). Shape analysis based on normalized principal moments of inertia reveals a distribution of conformations transitioning from elongated to more spherical forms with decreasing R_g_. Overall, peptide A5 adopts an unstructured and flexible conformation in solution, with local turn-like motifs. Details of the analysis are provided in the Supporting Information. In the subsequent phase of the study, averaged conformations of peptide A5 from family 1 were docked to the HVEM using the protein structure derived from the CD160/HVEM complex (PDB code: 6NG3). Docking was performed using the HDOCK platform. All ten top-ranked models demonstrated high confidence scores (>0.85). One of these models displayed a 3D structure similar to the fragment 110–128 of CD160 (Figure 5A) and bound to the CRD1 domain of HVEM, suggesting potential competitive interactions with BTLA. However, this model binds to the HVEM protein in a manner different from the corresponding fragment in the CD160 protein (Figure 5B). The remaining models primarily bound to the CRD2 of HVEM, and to a lesser extent to CRD3, rather than to the BTLA-binding CRD1 (Figure S12). The presence of high-confidence alternative binding modes suggests that peptide A5 may interact with additional functional sites on the HVEM surface. Conformational changes of the A5 peptide before and after binding to the HVEM protein, as well as the entropy contribution upon binding, were analyzed and are presented in Tables S6 and S7.

**Figure 5:**
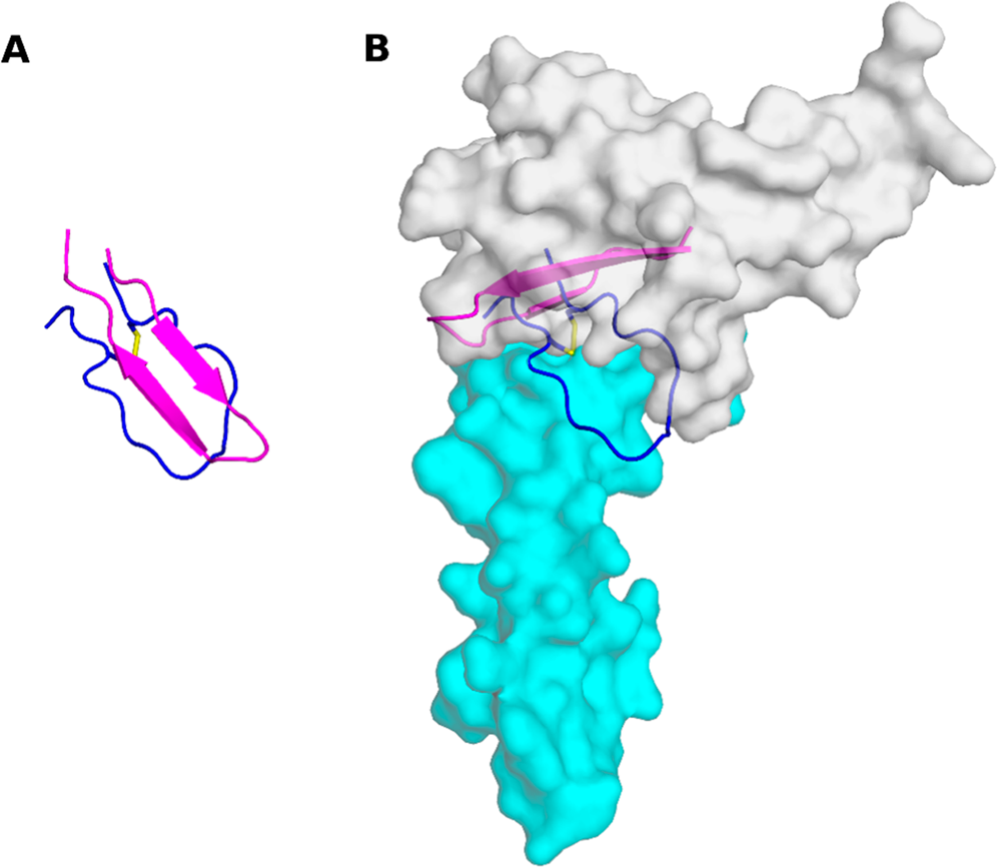
(A) The structural comparison of the CD160 fragment (residues 110–128) from the CD160/HVEM complex (PDB code: 6NG3, magenta) and peptide A5, obtained from docking the NMR structure to HVEM using HDOCK (dark blue); (B) structural comparison of three binding modes to HVEM (cyan surface): the peptide A5 predicted by HDOCK (dark blue ribbon), the CD160 fragment (residues 110–128) from the CD160/HVEM complex (PDB code: 6NG3) (magenta cartoon), and BTLA from the BTLA/HVEM complex (PDB code: 2AW2) (gray surface). HVEM was used as the common reference for structural superposition. The disulfide bond is represented as yellow sticks.

For the model of peptide A5 obtained through HDOCK docking that binds to CRD1 of HVEM protein, MM-GBSA analysis was conducted to identify the amino acid residues critical for the interaction between the modeled peptide and HVEM. The results indicated that the most significant residues, based on per-residue energy decomposition, were C125 and F127. According to pairwise per-residue energy decomposition, the key residues included C112, C113, A114, R115, S116, Q117, K118, R122, L123, C125, H126, F127, and F128 (Figure S13). It should be noted that, in the molecular docking, only natural amino acids were used; therefore, 2-aminobutanoic acid was substituted with a reduced cysteine residue.

An alanine scan of this peptide was also performed^38^ using FoldX,^39^ MM-GBSA,^40^ and PRODIGY,^41^ which confirmed that cysteine residues forming disulfide bonds are critical for peptide stability. It was suggested by FoldX that the substitutions Y110A (ΔΔG = −1.03 kcal/mol) and H126A (ΔΔG = −2.67 kcal/mol) could potentially enhance binding to HVEM. In contrast, no alanine substitutions were predicted by either MM-GBSA or PRODIGY to favorably affect binding, consistent with the overall energy decomposition results (Table S8).

### Inhibitory Properties of the CD160-Derived Peptides toward HVEM/LIGHT Complex

The molecular docking results for peptide A5 with the HVEM protein revealed that it interacts not only with the CRD1 but also with CRD2 and CRD3 of HVEM. These findings prompted us to investigate whether the peptide is also capable of inhibiting the binding of HVEM to its ligand—the LIGHT protein. Accordingly, ELISA and cell-based assays were conducted under the same conditions as those used for the BTLA/HVEM complex, with the exception that LIGHT was used instead of BTLA. The results demonstrate that peptide A5 inhibits not only the BTLA/HVEM interaction but also the HVEM/LIGHT complex formation. In ELISA, the peptide exhibited inhibitory effects of 89% at a concentration of 150 μM, 55% at 50 μM, and 13% at 16.67 μM (Figure 6A). Similarly, cell-based assays showed a significant reduction in NF-κB activation, with inhibition levels of 72%, 34%, and 13% at the corresponding concentrations (Figure 6B).

**Figure 6:**
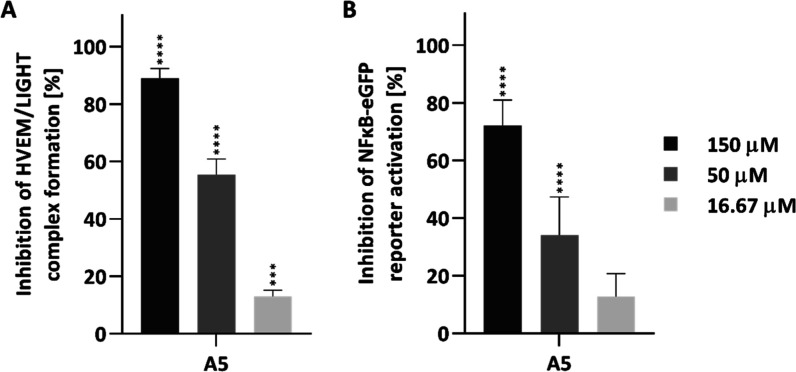
Inhibitory properties of peptide A5 toward the HVEM/LIGHT complex formation determined using (A) ELISA, (B) cellular-based assays. Results are shown for three experiments. Data are depicted as mean with SD. Statistical analysis was performed using one-way analysis of variance (ANOVA) followed by Dunnett’s post hoc test. ****p < 0.0001, ***p < 0.001, **p < 0.01, *p < 0.05.

## Discussion

The interaction between BTLA and HVEM negatively regulates the T cell-dependent immune response; thus, compounds targeting these proteins are still being sought. Anti-HVEM and anti-BTLA monoclonal antibodies (mAbs) are currently undergoing clinical trials for the treatment of both cancer and autoimmune diseases.^42^ Despite their exceptionally high efficacy, mAbs also present certain drawbacks, the most significant being the high cost associated with their development and production,^43^ as well as immune-related adverse events (irAEs). Although most irAEs present with mild to moderate symptoms, severe cases can lead to irreversible organ damage and pose an immediate threat to life.^44^ Currently, small-molecule compounds that inhibit the BTLA/HVEM complex formation and could serve as an alternative to antibodies have not been described. For many years, our research has focused on the development of peptide inhibitors targeting the BTLA/HVEM complex through rational peptide design. Until now, we have relied on the crystal structure of both proteins and their complexes with other molecules. In our previous studies, we designed a series of peptides targeting BTLA, based on fragments of the HVEM protein.^45,46^ One of them, referred to as HVEM(14–39), inhibits the BTLA/HVEM interaction and possesses immunomodulatory potential. In vitro studies conducted on T cells from healthy donors and melanoma patients demonstrated that it affects various T cell functions, including activation, proliferation, memory cell formation, apoptosis, and cytokine secretion.^47^

In our earlier research, we also designed peptides targeting HVEM, based on BTLA^33^ and gD^35,36^ binding fragments. Among the BTLA-derived peptides, two, referred to as BTLA(35–43) and BTLA(33–64)^C58Abu^, demonstrated blocking capacity, yet their effects on T cells have not been investigated.^33^ Several peptides based on the amino acid sequence of gD inhibited BTLA/HVEM binding; however, significant immunomodulatory potential was observed for only one, referred to as gD(1–36)­(K10C–D30C), which increased both the number of activated T lymphocytes and their proliferation.^48^ These and other findings suggest that the peptides may serve as alternatives to antibodies and are being extensively explored as immune checkpoint inhibitors.^49,50^ In the present study, we used another HVEM-binding protein, CD160, to design peptide inhibitors of BTLA/HVEM binding. CD160 interacts with HVEM at the same sites as BTLA; therefore, we investigated whether peptides based on the CD160 binding fragment could inhibit BTLA/HVEM complex formation.

The crystal structure of the CD160/HVEM complex reveals that amino acid residues Q111–R115, forming the F strand, and S119–F127, located within the G^0^ and G strands, constitute the core of the interaction with HVEM. These regions form numerous main-chain-to-main-chain hydrogen bonds, which significantly contribute to the overall stability of the protein complex. In the crystal structure, residues G120, I121, R122, L123, Q124, H126, and F127 of CD160 are crucial for the interaction with HVEM,^29^ which was confirmed by our MM-GBSA analysis results. Moreover, Liu et al. developed and evaluated a series of CD160 mutants. Their studies demonstrated that the I121N and R122W mutations in CD160 completely abolished binding to HVEM, while I121A and R122L mutants exhibited markedly reduced affinity. Furthermore, substitutions such as C113A, R115A, R115N, and H126A led to decreased binding, whereas mutations K118A, L123A, and Q124A had a less pronounced effect. Notably, none of the C-terminal mutants exhibited enhanced binding to HVEM compared to wild-type CD160.^29^ The results of MM-GBSA analysis indicate that residues C113, R115, and K118 are not critical for CD160/HVEM interaction (Figures S1A and S2A).

Amino acids S65, G66, and D67 in CD160 were also identified as important for interaction with HVEM. These residues are located within the CC’ loop, which is stabilized by a disulfide bond between cysteines at positions 61 and 68. Although the crystal structure suggests that these residues do not form direct contact with HVEM, they appear to play a role in maintaining the structural integrity of CD160.^29^ Interestingly, MM-GBSA analysis revealed interactions between S65 in CD160 and D7 in HVEM, as well as between G66 in CD160 and S20 in HVEM. Further mutational analysis of the CC′ loop and the C′ strand highlighted their functional relevance: single mutations such as D63A, E71A, and R78A in CD160 significantly impaired HVEM binding, while substitutions D67A and D67R resulted in a complete loss of interaction.^29^

In fragment 110–128 of CD160, which was used to design group A of peptides, two cysteine residues are present in positions 112 and 113. The former creates a disulfide bond with C44 in the native protein, while the latter remains unbound. In the designed peptides, these cysteines were substituted by 2-aminobutanoic acid to abrogate disulfide bond formation. The first designed peptide, A1, is linear and contains all amino acids from this region that are important for interaction with HVEM. Peptides A2-A5 contain disulfide bonds, which were introduced into the sequence of peptides by replacement of selected residues with cysteines. In peptide A2, F127—an amino acid important for protein binding, was replaced by cysteine. In peptides A3 and A4, residues Q124 and L123, respectively, both involved in CD160 binding to HVEM, were substituted with cysteines. Peptide A5 includes all amino acid residues identified as crucial for HVEM interaction, based on MM-GBSA analysis, and features a disulfide bond between positions 112 and 125. ELISA and cellular-based assays demonstrated that A5 was the only peptide in this group capable of disrupting the BTLA/HVEM complex formation (Figures 3 and 4). The lack of inhibitory activity observed for the remaining peptides indicates that both the presence of key binding residues and the proper positioning of the disulfide bond are essential for effective interaction with HVEM.

Subsequently, we designed the peptides that cover the middle part of the CD160 protein. To stabilize the structure of the CC’ loop, the peptides were extended with amino acids from the adjacent C and C′ strands. Group B peptides cover residues 56–79, while group C peptides include the shorter fragment comprising residues 59–75. Peptides B1 and C1 are linear, with both cysteines substituted by 2-aminobutanoic acid. In the remaining peptides, disulfide bonds were introduced by replacing selected residues with cysteines: V57 and R78 in B2, L60 and L74 in B3, and F59 and Q76 in B4. Notably, none of these substituted residues are directly involved in HVEM binding. Peptide B5 contains a disulfide bond between residues C61 and C68, which naturally occurs in the native CD160 protein. Peptide C2 is a truncated version of B3, while C3 corresponds to B5. ELISA and cell-based assays revealed that none of the peptides from groups B and C significantly inhibited protein binding. Some inhibitory properties were observed for peptides B1 and B2 in ELISA, at a concentration of 150 μM (Figure 3); however, in cellular assays, only peptide B1 showed a weak blocking effect, as peptide B2 exhibited cytotoxicity at this concentration (Figures 4 and S5).

In the next stage of our study, we conducted an in-depth conformational analysis of peptide A5, the most effective inhibitor of BTLA/HVEM binding. The results revealed that despite the introduction of a disulfide bond intended to stabilize its structure, the peptide remains highly flexible and does not adopt a well-defined tertiary conformation (Figure S11). Molecular docking analysis further demonstrated that peptide A5 binds not only to the CRD1 of HVEM, which is responsible for interaction with BTLA, but also to the CRD2 and CRD3 (Figure S12). The binding of the peptide to the HVEM is also different from that of the corresponding fragment in the CD160 protein. Peptide A5 displays structural flexibility, enabling it to adjust its conformation upon binding to HVEM. This feature results in an expanded binding interface, as evidenced by MM-GBSA analysis, which shows that more residues from the 110–128 region are involved compared to CD160. In the CD160/HVEM complex, the main contribution was observed for CD160 residues G120 to F127 (excluding G125), as revealed by both types of energy decomposition analysis (Figures S1A and S2A). In contrast, in the HVEM/A5 complex, nearly all residues covering the 110–128 fragment of the peptide contributed to binding, except for Y110, Q111, G120, I121, and Q124, according to pairwise per-residue energy decomposition. However, based on per-residue energy decomposition, only two residues, C125 and F127, appear to be significant (Figure S13).

Based on molecular docking results indicating that the peptide A5 engages not only the CRD1 domain but also CRD2 and CRD3 of HVEM, we hypothesized that it might also interfere with the HVEM/LIGHT interaction. To explore this possibility, we conducted further ELISA and cell-based assays, which confirmed that peptide A5 inhibits both BTLA/HVEM and HVEM/LIGHT interactions (Figure 6). These findings are fully consistent with the molecular docking data and support the conclusion that A5 engages multiple domains of HVEM, making it a potential dual-function inhibitor with broader immunomodulatory potential.

The ability of peptide A5 to simultaneously disrupt both BTLA/HVEM and HVEM/LIGHT interactions may have significant therapeutic implications in the context of immune-related diseases. These two pathways are known to play opposing roles in immune regulation: BTLA/HVEM signaling acts as an inhibitory checkpoint that limits T cell activation,^2^ while HVEM/LIGHT activates immune responses.^51^ In autoimmune diseases, where immune responses are overactive, inhibition of the HVEM/LIGHT signaling pathway could help dampen inflammation. At the same time, targeting the BTLA/HVEM interaction could interfere with inhibitory signals essential for maintaining self-tolerance. Conversely, in cancer, where immune responses are often suppressed, simultaneous blockade of both pathways may enhance antitumor immunity by suppressing inhibitory BTLA-mediated signaling and by disrupting pro-tumor HVEM-mediated signaling within the tumor microenvironment.^27,28,52^ Thus, peptide A5 represents a promising dual-targeting molecule with potential applications in both autoimmunity and oncology. However, its dual activity also underscores the need for context-specific evaluation, as modulation of these pathways may yield divergent effects depending on the disease’s immune landscape. Further, in vitro and in vivo studies will be crucial in determining its therapeutic value and safety profile.

## Conclusion

Our research focuses on identifying inhibitors of the BTLA/HVEM protein complex formation. In this study, we designed a series of peptides based on the amino acid sequence of CD160, binding to HVEM. These peptides were subsequently evaluated for their affinity to HVEM and their capacity to inhibit the BTLA/HVEM interaction. Among them, peptide A5 demonstrated strong binding to HVEM and exhibited the most potent inhibitory activity. Notably, further analyses revealed that A5 also inhibits the HVEM/LIGHT complex formation, suggesting its potential as a dual-function inhibitor. As the next step in our research, it will be essential to investigate the immunomodulatory effects of peptide A5 on T cell activation in both cancer patients and individuals with autoimmune diseases. Furthermore, comprehensive in vivo studies will be necessary to evaluate its therapeutic potential and safety profile.

## Materials and Methods

### Sources of Proteins

Recombinant human HVEM protein with a His tag was purchased from ACROBiosystems, Newark, DE, USA (no. HVM-H52E9). Recombinant human protein BTLA-Fc and LIGHT-Fc were also purchased from ACROBiosystems, Newark, DE, USA (no. BTA-H5255 and no. LIT-H5269).

### Molecular Dynamics and MM-GBSA Analysis

Analyses were conducted based on the crystal structure of the CD160/HVEM protein complex (PDB code: 6NG3). To determine the most strongly interacting residues, the simulations of the whole protein complex were performed. The input files necessary for the simulation were prepared on the basis of the tleap program using the ff19SB force field with a four-site OPC water model.^53–55^ The simulations were run using pmemd.cuda from the AMBER22 package.^53^ The simulated systems were surrounded by a 17.5 Å layer of water in the shape of a truncated octahedron, and the total charge of the system was neutralized by the addition of Na^+^ or Cl^–^. The systems underwent two cycles of minimization, 1000 steps with restraints on each atom not belonging to the solvent molecule, and 2000 steps with restraints on all heavy atoms. This was followed by three equilibrating cycles—the first to heat the system from 10 to 300 K over 10,000 MD steps, the second of 100,000 steps to achieve the present density. Finally, a third equilibration consisting of 400,000 steps npt with a Langevin thermostat^40,56^ and weak restraints on Cα and Cβ atoms was performed to ensure system stability. On the basis of such a prepared system, three independent molecular dynamics simulations were performed, each with 100,000,000 steps with a length of 2 fs, which corresponds to 200 ns of simulation each. Analyses were performed on the last 20 ns of the 200 ns simulation to minimize the potential impact of the simulation origins on the convergence, the convergence was monitored using collective variables^57^ (the changes in Rg, Rgmax and RMSD for A5, CD160, HVEM/A5 and CD160/HVEM during the simulation are presented in Figures S14–S17). To estimate the binding free energy, the MM-GBSA method, with the GB-Neck2 estimation method and the associated radii values, was used.^58–60^ Per-residue and pairwise per-residue energy decomposition, to determine the interaction energy of individual residues and their contribution to the CD160/HVEM and HVEM/A5 complexes binding energy, was performed. Moreover, the entropic contribution to the binding free energy (TΔS) of the HVEM/A5 complex was calculated using normal-mode analysis for 10 frames uniformly extracted from the last 20 ns of the 200 ns simulations (Table S7).^61^

### Peptide Synthesis

All peptides were synthesized using the solid-phase peptide synthesis (SPPS) method and the Fmoc/*t*Bu strategy. The synthesis was carried out using LibertyBlue microwave-assisted automated peptide synthesizer (CEM Corporation, Matthews, NC, USA) and Rink Amide Pro Tide (LL) resin, with a loading of 0.18 mmol/g, which was used as the solid phase. Prior to synthesis, amino acids were dissolved in *N,N*-dimethylformamide (DMF) to a final concentration of 0.2 M. To deprotect the N-terminal amine group of the peptide, 20% piperidine in DMF was used, while the activation of the C-terminal carboxylic group of the attached amino acid was achieved with a 1 M solution of ethyl 2-cyano-2-(hydroxyamino)­acetate (Oxyma Pure) in DMF. As the coupling agent, a 0.5 M solution of *N*,*N*′-diisopropylcarbodiimide (DIC) in DMF was used. The N-terminal amino group of the peptide was acetylated by dissolving *N*-acetylimidazole (1.1 g per 1 g of resin) in 15 mL of DMF, and stirring for 24 h with peptidyl-resin. In the next step, the peptide was cleaved from the resin. A mixture containing 17.6 mL of trifluoroacetic acid (TFA), 1 mL of phenol, 1 mL of water, and 0.4 mL of triisopropylsilane (TIPS) was added to the peptidyl-resin and stirred for 4 h. The resulting solution was then filtered through a Schott funnel, and cold diethyl ether was added to precipitate the peptide. The precipitate was centrifuged at 2773 × g (4000 rpm) for 15 min at 4 °C and then allowed to dry completely. Finally, the peptide was dissolved in water and lyophilized.

### Peptide Purification

The peptides were purified using reversed-phase high-performance liquid chromatography (RP-HPLC) with a UV detector (Knauer, Berlin, Germany) on a Luna C8(2) semipreparative column (21.2 × 250 mm, 5 μm) (Phenomenex, Torrance, CA, USA). Initially, the peptides were dissolved in H_2_O, and if they contained cysteine residues with free –SH groups, a 10-fold molar excess of dithiothreitol (DTT) was added to the solution. Purification was carried out using a linear gradient of solution B (80% acetonitrile in water with 0.08% TFA) in solution A (water with 0.1% TFA), individually optimized for each peptide. The purity of individual fractions was verified using an LC ESI-IT-TOF MS instrument and reversed phase-ultra high performance liquid chromatography (RP-UHPLC) with ELSD-LT and PDA detectors (Shimadzu, Kyoto, Japan) on a Kinetex C8 analytical column (2.1 × 100 mm, 2.6 μm) (Phenomenex, Torrance, CA, USA). The analysis was performed using a linear gradient from 5% to 100% of solution B in solution A, in a 15 min time frame. After obtaining pure fractions, acetonitrile was evaporated and the peptides were lyophilized.

### Formation of Disulfide Bond

A disulfide bond was formed by dissolving the peptide in a water/methanol solution at a ratio of 1:9 (v/v), with a maximum peptide concentration of 50 mg/L. The pH of the mixture was adjusted to 8–9 using a 25% ammonia solution. The mixture was stirred at room temperature for 7 days, during which time compressed air was introduced into the solution. After this period, the solvents were evaporated. To confirm the oxidation of the cysteine residues, RP-UHPLC analysis was performed. The product was subsequently lyophilized and further purified using RP-HPLC, as previously described. All compounds are >95% pure by RP-UHPLC analysis.

### Spectral Shift

All samples for SpS were prepared using Monolith His-tag Labeling Kit RED-tris-NTA second Generation (no. MO-L018, NanoTemper Technologies GmbH, Munich, Germany), and measurements were conducted on Monolith X (NanoTemper Technologies GmbH, Munich, Germany). PBS containing 140 mM sodium chloride, 2.7 mM potassium chloride, and 10 mM phosphate buffer, pH 7.4, was used as the assay buffer. The HVEM-His protein was labeled with RED-tris-NTA second Generation dye by mixing 100 nM HVEM-His with 100 nM dye and incubating the mixture for 30 min at room temperature. Peptides were initially dissolved in PBS to prepare 500 μM stock solutions, followed by serial dilutions in PCR tubes using PBS to generate 16 measurement points. Subsequently, the labeled HVEM protein was added to each dilution, resulting in a final protein concentration of 25 nM and peptide concentrations ranging from 7.6 nM to 250 μM. Each sample was transferred to a standard glass capillary (no. MO-K022, NanoTemper Technologies GmbH, Munich, Germany) and loaded to the Monolith X. Measurements were performed at 25 °C under the following conditions: response evaluation mode—670 nm/650 nm ratio, excitation power—100%, IR laser power—high. The data were visualized using GraphPad Prism 8 software (San Diego, CA, USA).

### Enzyme-Linked Immunosorbent Assays

ELISA was performed using transparent 96-well NUNC Maxisorp plates (Thermo Fisher Scientific, Waltham, MA, USA). In the first step, HVEM-His, dissolved in PBS at a concentration of 5 μg/mL (100 μL/well), was immobilized on the plate by overnight incubation at 4 °C. Wells were then blocked with 5% bovine serum albumin (BSA) in PBS containing 0.05% Tween 20 (PBS-T) (200 μL/well). Next, peptides dissolved in PBS-T at concentrations of 150, 50, and 16.67 μM were added to the wells (100 μL/well). Following this, BTLA-Fc in PBS-T at a concentration of 5 μg/mL (100 μL/well) was applied. In the next step, goat anti-human IgG antibody conjugated with the HRP (Bio-Rad Laboratories, Hercules, CA, USA) in PBS-T at a ratio of 1:30,000 (v/v) was added (100 μL/well). Colorimetric detection was performed by adding 100 μL of TMB substrate (Thermo Fisher Scientific, Waltham, MA, USA) to each well and incubating the plate in the dark. At each stage, plates were incubated at 37 °C with continuous shaking for 1 h (except TMB, which was incubated for 15 min). After each incubation, wells were washed five times with PBS-T. Absorbance was measured using an Infinite M200 Pro Reader (Tecan Group Ltd., Männedorf, Switzerland) at wavelengths of 650 nm (measurement wavelength) and 492 nm (reference wavelength). Inhibition assays for the HVEM/LIGHT complex were conducted using the same approach, except that LIGHT was used instead of BTLA. The BTLA/HVEM and HVEM/LIGHT complexes without peptides served as controls. Inhibition percentages were calculated based on the assumption that PBS-T has no effect on the formation of BTLA/HVEM or HVEM/LIGHT complexes. Each ELISA was conducted in at least three independent replicates. The data were analyzed using GraphPad Prism 8 software (San Diego, CA, USA).

### Cell Cultures

The Jurkat E6.1 cells were purchased from CLS Cell Lines Service GmbH (Eppelheim, Germany), while the generation of the HVEM (JE6.1-NF-κB::eGFP) cell line and the T cell stimulator (TCS and TCS BTLA) have been described previously.^37,62,63^ All cell lines were cultured in RPMI 1640 supplemented with 10% FBS, penicillin (100 U/mL), and streptomycin (100 μg/mL) (Sigma-Aldrich, St. Louis, MO, USA), and incubated at 5% CO_2_ atmosphere at 37 °C.

### Cellular-Based Assay

Cellular studies were performed in the same procedure as we described previously for gD- and BTLA-derived peptides.^33,35,36^ The peptides were dissolved in the medium, and their final concentrations in wells were 150, 50, and 16,7 μM. Briefly, reporter cells expressing HVEM (JE6.1-NF-κB::eGFP) (5 × 10^4^ cells/well) were preincubated with peptides for 30 min in a 96-well flat-bottom plate. Subsequently, the second cell line expressing BTLA (TCS BTLA) or LIGHT (TCS LIGHT) (2 × 10^4^ cells/well) was added and cocultured for 24 h at 37 °C in an atmosphere of 5% CO_2_. Next, an APC-conjugated mCD45.2 antibody to exclude TCS from the reporter cells was added. The expression of the reporter gene (eGFP) was measured via flow cytometry using a CytoFLEX flow cytometer (Beckman Coulter Inc., Brea, CA, USA), and the results were analyzed using FlowJo v10.10 (FlowJo LLC, Ashland, OR, USA). The data were analyzed using GraphPad Prism 8 software (San Diego, CA, USA).

### NMR Measurements

NMR spectra were acquired at 298 K on Bruker spectrometers (^1^H 700.55 MHz, 16.4 T and 500.13 MHz, 11.7 T). The following NMR experiments were collected with a sample of 2 mM in H_2_O/D_2_O (9:1, v/v): 1D ^1^H, TOCSY (τ_mix_ = 80 ms), NOESY (τ_mix_ = 100 and 200 ms), and ^1^H–^13^C HSQC. The ^1^H–^13^C HSQC spectrum was recorded at the natural abundance of the ^13^C isotope. Chemical shifts were referenced to external sodium 2,2-dimethyl-2-silapentane-5-sulfonate (DSS), with Ξ = 0.251449530 used for indirect referencing of ^13^C resonances.^64^ Spectra were processed and analyzed, respectively, with Topspin 4.4.1 (Bruker, Ettlingen, Germany) and NMRFAM-Sparky^65^ software packages. Distance constraints used in the structure determination were derived from the NOESY spectra with the mixing time of 200 ms.

### Molecular Modeling

Molecular dynamics simulations were conducted using the ff19SB force field within the AMBER 24 package.^40^ The three-dimensional structures of the peptide were generated through a simulated annealing (SA) approach, incorporating time-averaged interproton distance restraints. These distances were derived from NOE intensities using the CALIBA algorithm in CYANA 2.1.^66^ The simulated annealing procedure began from a random conformation, applying time-averaged interproton distance restraints with a force constant of 50 kcal/(mol × Å^2^). The peptide backbone geometry was maintained in the trans configuration, in accordance with NMR data (50 kcal/(mol × rad^2^)). A total of 1000 SA cycles were performed, where each conformation served as the starting point for the next cycle. Each cycle comprised 30,000 molecular dynamics steps (30 ps), including heating to 1200 K over 1 ps, equilibration at 1200 K for 2 ps, and gradual cooling to 0 K over 27 ps. Solvent effects were approximated using the generalized Born implicit solvent model.^59^ To assess agreement with experimental data, deviations from interproton distance restraints were evaluated using the SANDER module in AMBER 24. Conformations with a total distance penalty of ≤0.5 kcal/mol were selected and subjected to energy minimization without constraints. These structures were then categorized into conformational families using hierarchical agglomerative clustering within the CPPTRAJ module. The principal moments of inertia (I_1_, I_2_, and I_3_) were computed using a standard tool of the GROMACS 2019 package,^67^ and molecular shape analysis was performed based on normalized PMI ratios (I_1_/I_3_ and I_2_/I_3_) for each structure. Finally, conformational visualization was carried out using VMD 1.9.4a38.^68^

### Peptide Docking in HDOCK

The NMR structure of the most promising peptide A5 was determined, and the structural family with the highest probability was selected from the ensemble. The average structure of this family was then calculated using cpptraj by averaging atomic coordinates. This average structure was then subjected to short minimization (500 steps steepest-descent followed by 500 steps of conjugated gradient) without any restraints using the SANDER program from the AMBER package.^69^ The minimized structure was then subjected to docking to the HVEM protein using HDOCK.^70^ All 10 complex models exhibited a high confidence score ranging from 0.86 to 0.90. The model of peptide binding to HVEM at the same site as BTLA was selected for molecular dynamics simulations (DOI: 10.71853/hwrc-3p52). The protocol for MD simulations was the same as in the case of the whole CD160/HVEM complex.

## Supplementary Material





## Data Availability

Structure of HVEM/A5 peptide: doi: 10.71853/hwrc-3p52.

## Notes

Per-residue and pairwise per-residue MM-GBSA energy decomposition analyses for CD160/HVEM and HVEM/A5 peptide complexes; decomposition of binding free energy contributions; experimental and theoretical binding affinities for BTLA/HVEM and HVEM/peptide complexes; cytotoxicity and stability studies of the A5 peptide; CD and NMR characterization of the A5 peptide, including conformational clustering and secondary structure analyses; and molecular docking of peptide A5 with the HVEM protein (PDF) (PDF)
Molecular formula strings and corresponding biological data (CSV file) (CSV)

## Abbreviations

aCD3: anti-human CD3 single-chain antibody fragment
BTLA: B- and T-lymphocyte attenuator
CD3: cluster of differentiation 3
CD160: cluster of differentiation 160
CRD: cysteine-rich domain
eGFP: enhanced green fluorescent protein
gD: glycoprotein D
HCMV: human cytomegalovirus
HRP: horseradish peroxidase
HSV-1/2: herpes simplex virus type 1/2
HVEM: herpes virus entry mediator
ICP: immune checkpoint
IgSF: immunoglobulin superfamily
irAE: immune-related adverse event
ITIM: immunoreceptor tyrosine-based inhibitory motif
ITSM: immunoreceptor tyrosine-based switch motif
K_d_: dissociation constant
LIGHT: homologous to lymphotoxin, exhibits inducible expression, and competes with HSV glycoprotein D for herpes virus entry mediator, a receptor expressed by T lymphocyte
LTα: lymphotoxin α;
mAb: monoclonal antibody
MM-GBSA: molecular mechanics-generalized Born surface area
NF-κB: nuclear factor kappa light chain enhancer of activated B cell
NK: natural killer cell
NOE: nuclear Overhauser effect
NOESY: nuclear Overhauser effect spectroscopy
R_g_: radius of gyration
SH2: Src homology 2 domain
SLE: systemic lupus erythematosus
SpS: spectral shift
SPPS: solid-phase peptide synthesis
TCR: T cell receptor
TCS: T cell stimulator
TIL: tumor-infiltrating lymphocyte
TMB: 3,3′,5,5′-tetramethylbenzidine
TNFRSF: tumor necrosis factor receptor superfamily
TOCSY: total correlation spectroscopy
Treg: regulatory T cell.
