# Supplementary material for: CD160-Derived Peptide as a Bidirectional Inhibitor Toward Immune Checkpoints BTLA/HVEM and HVEM/LIGHT
Source: J Med Chem. 2025 Nov 30;68(23):25078–90. doi: 10.1021/acs.jmedchem.5c02037 (PMC12714101; doi:10.1021/acs.jmedchem.5c02037)
Supplement: Supplementary file 2 [file jm5c02037_si_002.pdf]

Supporting Information

CD160-derived peptide as a bidirectional inhibitor towards immune checkpoints BTLA/HVEM and HVEM/LIGHT

Magdalena Lipińska[1], Piotr Ciura[1], Simon Gumpelmair[2], Emilia Sikorska[1], Katarzyna Kuncewicz[1], Adam K. Sieradzan[1], Peter Steinberger[2], Anna Wardowska[3], Marta Spodzieja[1*]

[1] University of Gdańsk, Faculty of Chemistry, Wita Stwosza 63, 80-308 Gdańsk, Poland
[2] Medical University of Vienna, Institute of Immunology, Division of Immune Receptors and T cell Activation, Lazarettgasse 19, 1090 Vienna, Austria
[3] Medical University of Gdańsk, Department of Rheumatology, Clinical Immunology, Geriatrics and Internal Medicine, Smoluchowskiego 17, 80-214 Gdańsk, Poland

*Corresponding author: Marta Spodzieja, marta.spodzieja@ug.edu.pl

Contents of SI

Figures:

Figure S1. Per-residue total energy decomposition analysis for A) CD160 and B) HVEM amino acid residues……………………………………………………………………………..**S3**

Figure S2. Pairwise per-residue total energy decomposition analysis for A) CD160 and B) HVEM amino acid residues…………………………………………………………………..**S4**

Figure S3. Per-residue energy decomposition divided into van der Waals + non-polar and electrostatic + polar contributions for A) CD160 and B) HVEM amino acid residues……...…**S6**

Figure S4. Pairwise per-residue energy decomposition divided into van der Waals + non-polar and electrostatic + polar contributions for A) CD160 and B) HVEM amino acid residues……**S7**

Figure S5. The influence of the CD160-derived peptides on the viability of A) Jurkat E6.1 and B) TCS cell lines after incubation for 24 hours……………………………………………**S10**

Figure S6. Stability of peptides in Jurkat E6.1 cell supernatant after 0 and 24 hours of incubation…………………………………………………………………………………....**S11**

Figure S7. Comparison of chromatograms obtained for: A) peptide A5 dissolved in H_2O at time 0; B) peptide A5 dissolved in cell culture supernatant at time 0; C) cell culture supernatant at time 0; D) peptide A5 in cell culture supernatant after 24 hours of incubation; E) cell culture supernatant after 24 hours of incubation……………………………………………….…….**S12**

Figure S8. CD spectra of the A5 peptide under various conditions: A) in H_2O and PBS, and depending on: B) ionic strength, C) PBS concentration, and D) PBS pH…………...……..**S14**

Figure S9. A) Overlay the HN-Hα region of the TOCSY (green-blue) and NOESY (red-yellow) spectra recorded for A5 at 298 K; B) Distribution of distance restraints……………………..**S17**

Figure S10. A) Sequence plots of NOESY distance constraints for A5; B) Hα and Cα chemical shift analysis……………………………………………………………………………**S18**

Figure S11. A) Superimposed conformations; B) Ramachandran plot; C) secondary structure analysis; only extended, turn, and bend structures were observed; other types were absent or negligible; and D) principal moment of inertia (PMI) plot illustrating the three-dimensional shape diversity of the conformations of peptide A5 from a dominant conformational family…………………………………………………………………………………..**S20**

Figure S12. Structural representation of the BTLA (grey surface)/HVEM (cyan surface) complex (PDB code: 2AW2) with the ten top-ranked peptide A5 binding modes predicted by HDOCK……………………………………………………………………………………..**S21**

Figure S13. A) Per-residue and B) pairwise per-residue energy decomposition analysis for peptide A5. The criterion for "strong" interaction energy was defined as an energy of -1 kcal/mol or lower for per-residue decomposition, and -3 kcal/mol or lower for pairwise per-residue decomposition……………………………………………………………………………**S22**

Figure S14. A) Rg, B) Rgmax, and C) RMSD for the A5 peptide during the simulation…….**S24**

Figure S15. A) Rg, B) Rgmax, and C) RMSD for the CD160 protein during the simulation……………………………………………………………………………………**S25**

Figure S16. A) Rg, B) Rgmax, and C) RMSD for the HVEM/A5 complex during the simulation……………………………………………………………………………………**S26**

Figure S17. A) Rg, B) Rgmax, and C) RMSD for the CD160/HVEM complex during the simulation……………………………………………………………………………………**S27**

Tables:

Table S1. The pairwise per-residue energy decomposition obtained for CD160/HVEM complex calculated using the MM-GBSA method…………………………………………………….**...S5**

Table S2. Decomposition of the binding free energy (ΔG) for the CD160/HVEM complex into van der Waals + non-polar and electrostatic + polar contributions obtained from MM-GBSA analysis……………………………………………………………………………………..**S5**

Table S3. Experimental and theoretical binding affinities for the BTLA/HVEM and HVEM/peptide complexes……………………………………………………………………**S8**

Table S4. Proton chemical shifts for A5 peptide at 298 K……………………………………**S16**

Table S5. Summary of conformational clusters found by using of a hierarchical agglomerative algorithm……………………………………………………………………………………**S19**

Table S6. Percentage of secondary structures calculated using the cpptraj algorithm for the A5 peptide based on molecular dynamics……………………………………………………**S21**

Table S7. Entropic contributions (TΔS) calculated from normal mode analysis (NMA) for HVEM in complexes with peptide A5 (obtained from CD160/HVEM complex and NMR)...**S22**

Table S8. Comparison between different methods of free energy change difference of alanine mutation (ΔΔG) obtained with FoldX, with the from MMGBSA alanine scan and from Prodigy………………………………………………………………………………………**S23**

MM-GBSA analysis

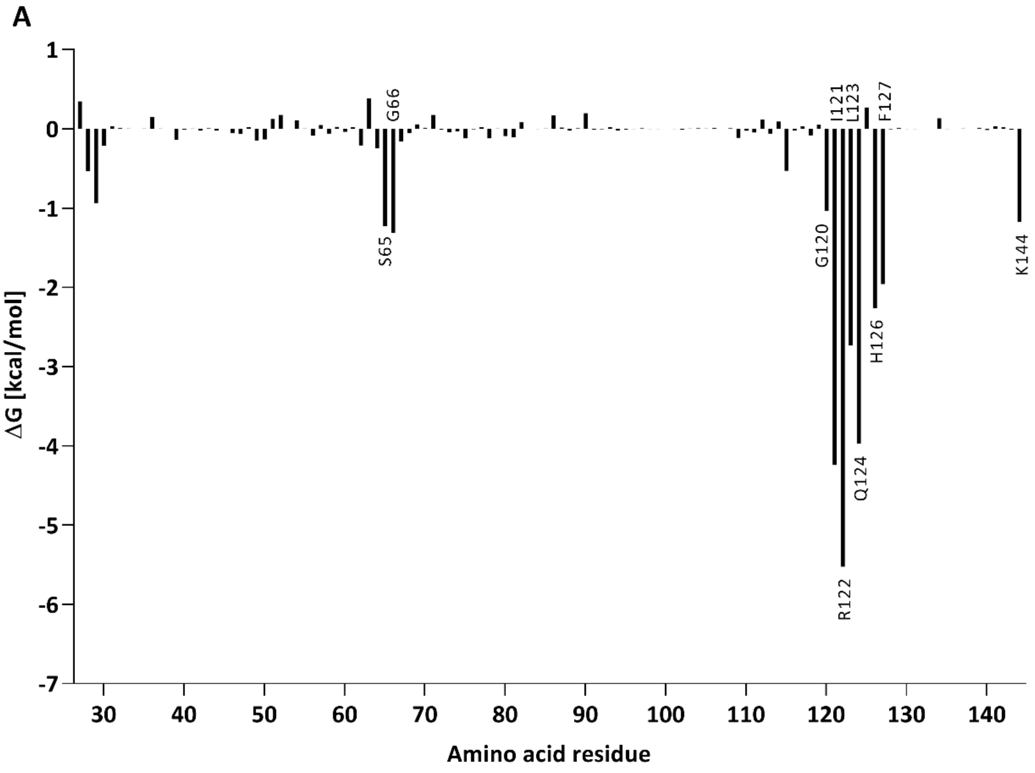

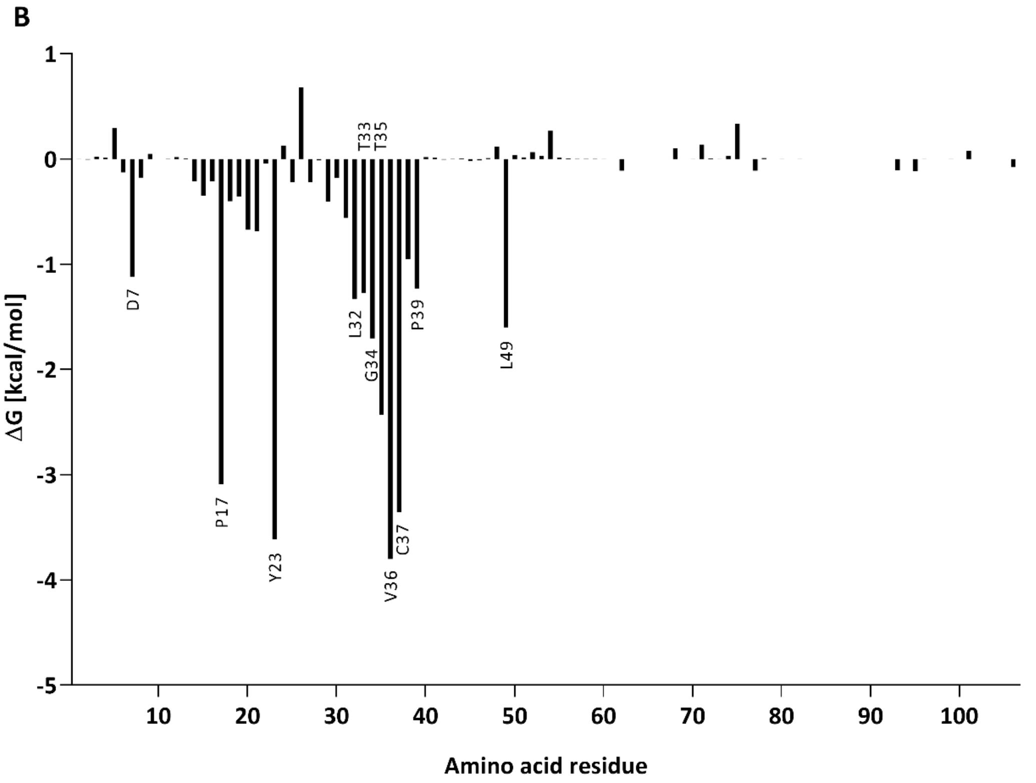

Figure S1. *Per-residue total energy decomposition analysis for A) CD160 and B) HVEM amino acid residues. The cut-off for a "strong" interaction (ΔG) was defined as -1 kcal/mol or lower.*

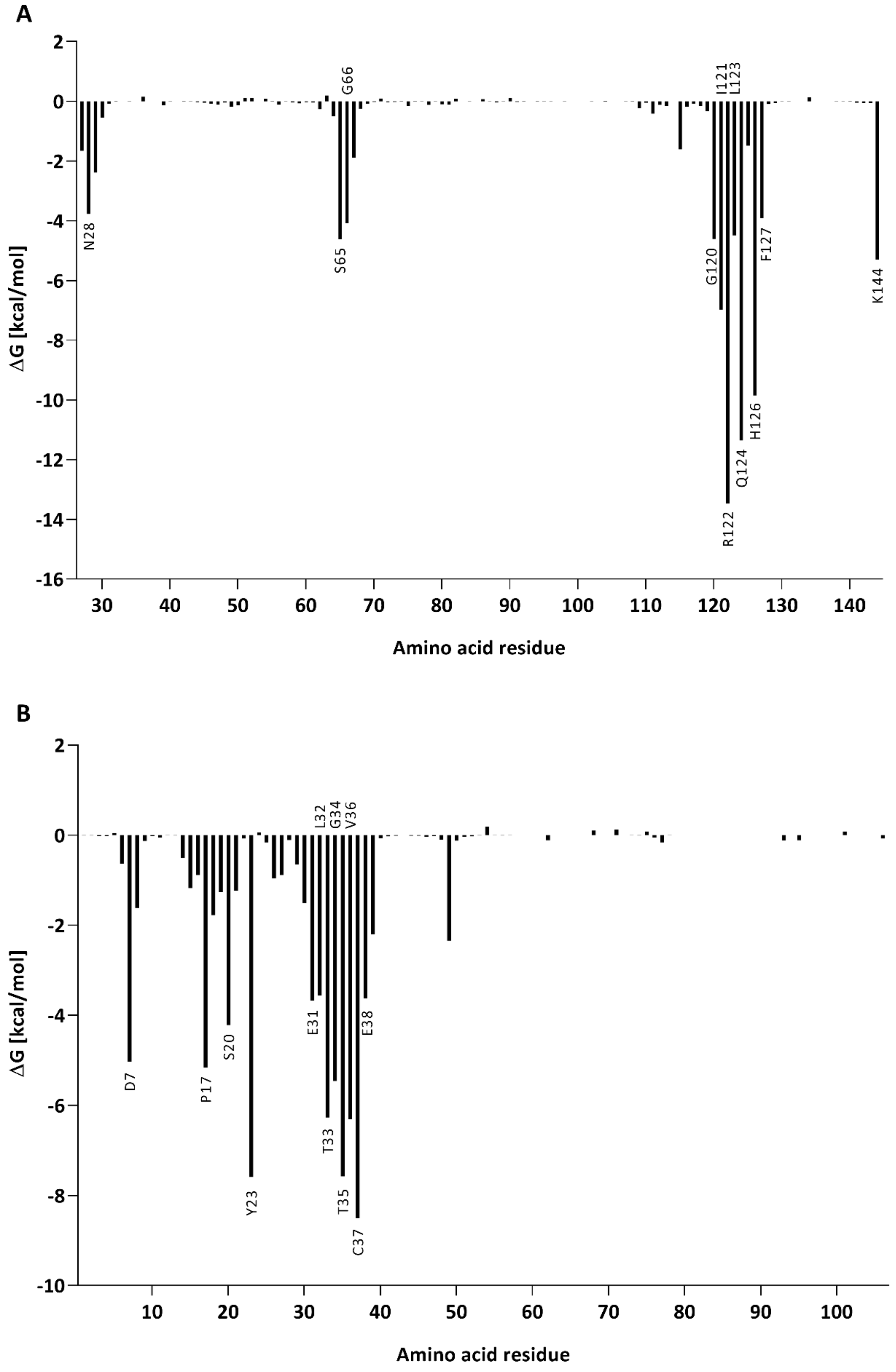

Figure S2. *Pairwise per-residue total energy decomposition analysis for A) CD160 and B) HVEM amino acid residues. The cut-off for a "strong" interaction (ΔG) was defined as -3 kcal/mol or lower.*

Table S1. *The pairwise per-residue energy decomposition obtained for CD160/HVEM complex calculated using the MM-GBSA method. The criterion for "strong" interaction energy was defined as -3 kcal/mol or lower.*

**Interaction energy [kcal/mol]**	**CD160 residue**	**HVEM residue**
-13.470	R122	C37
-11.358	Q124	T35
-9.847	H126	P17
-6.976	I121	C37
-5.230	K144	E31
-4.614	S65	D7
-4.608	G120	Y23
-4.485	L123	T35
-4.086	G66	S20
-3.907	F127	P17

Table S2. *Decomposition of the binding free energy (ΔG) for the CD160/HVEM complex into van der Waals + non-polar and electrostatic + polar contributions obtained from MM-GBSA analysis.*

<table>
<tr><th rowspan="2">Complex</th><th rowspan="2">Energy decomposition</th><th rowspan="2">Protein</th><th colspan="2">van der Waals + non-polar</th><th colspan="2">electrostatic + polar</th></tr>
<tr><th>ΔG [kcal/mol]</th><th>SD</th><th>ΔG [kcal/mol]</th><th>SD</th></tr>
<tr><td rowspan="4">CD160/HVEM</td><td rowspan="2">per-residue</td><td>CD160</td><td>-41.79</td><td>6.76</td><td>14.81</td><td>2.55</td></tr>
<tr><td>HVEM</td><td>-41.52</td><td>6.67</td><td>13.49</td><td>0.858</td></tr>
<tr><td rowspan="2">pairwise per-residue</td><td>CD160</td><td>-54.19</td><td>7.92</td><td>-33.65</td><td>10.54</td></tr>
<tr><td>HVEM</td><td>-52.82</td><td>7.47</td><td>-31.61</td><td>9.64</td></tr>
</table>

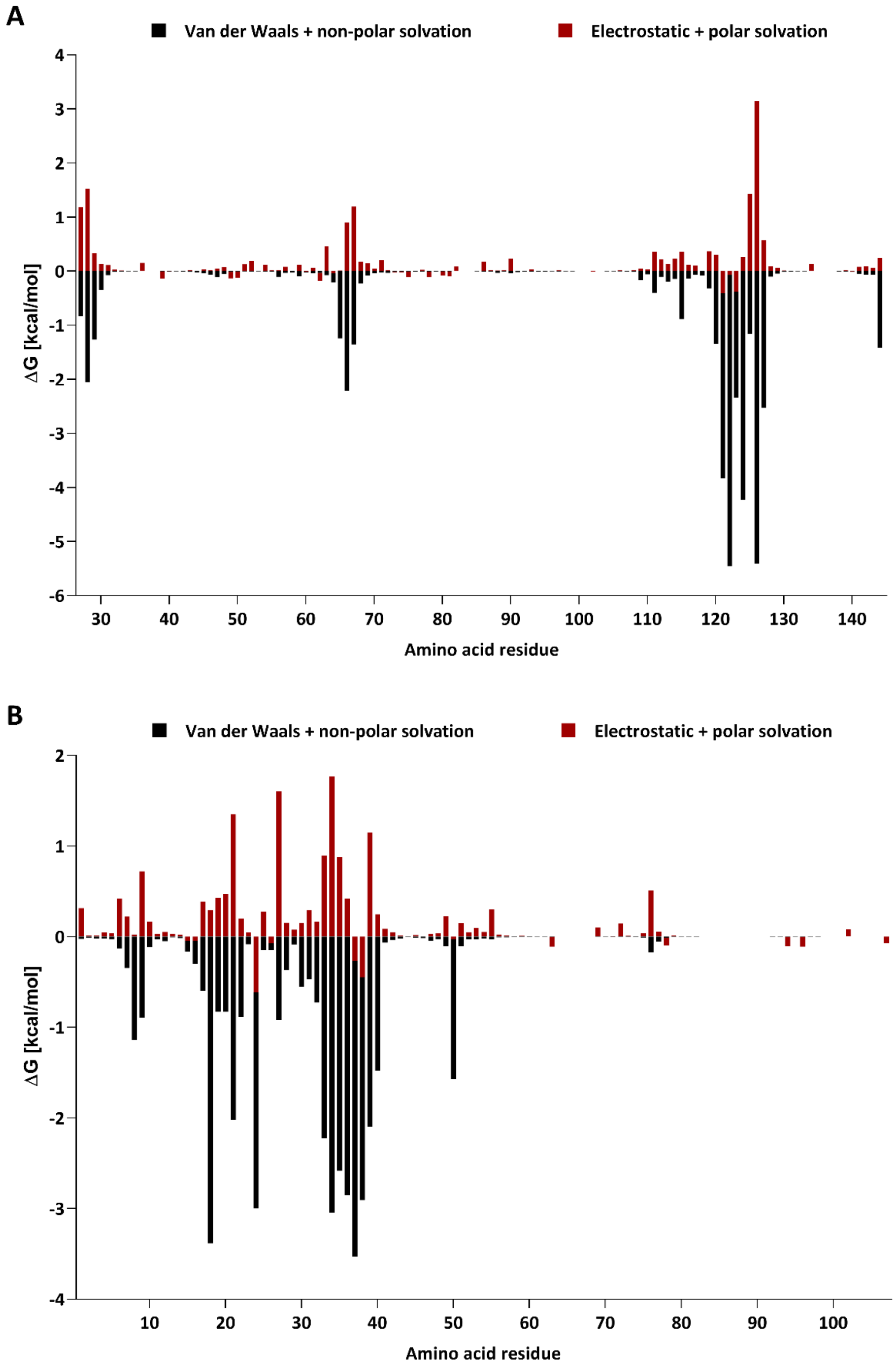

Figure S3. *Per-residue energy decomposition divided into van der Waals + non-polar and electrostatic + polar contributions for A) CD160 and B) HVEM amino acid residues. The energy values were superimposed for each residue.*

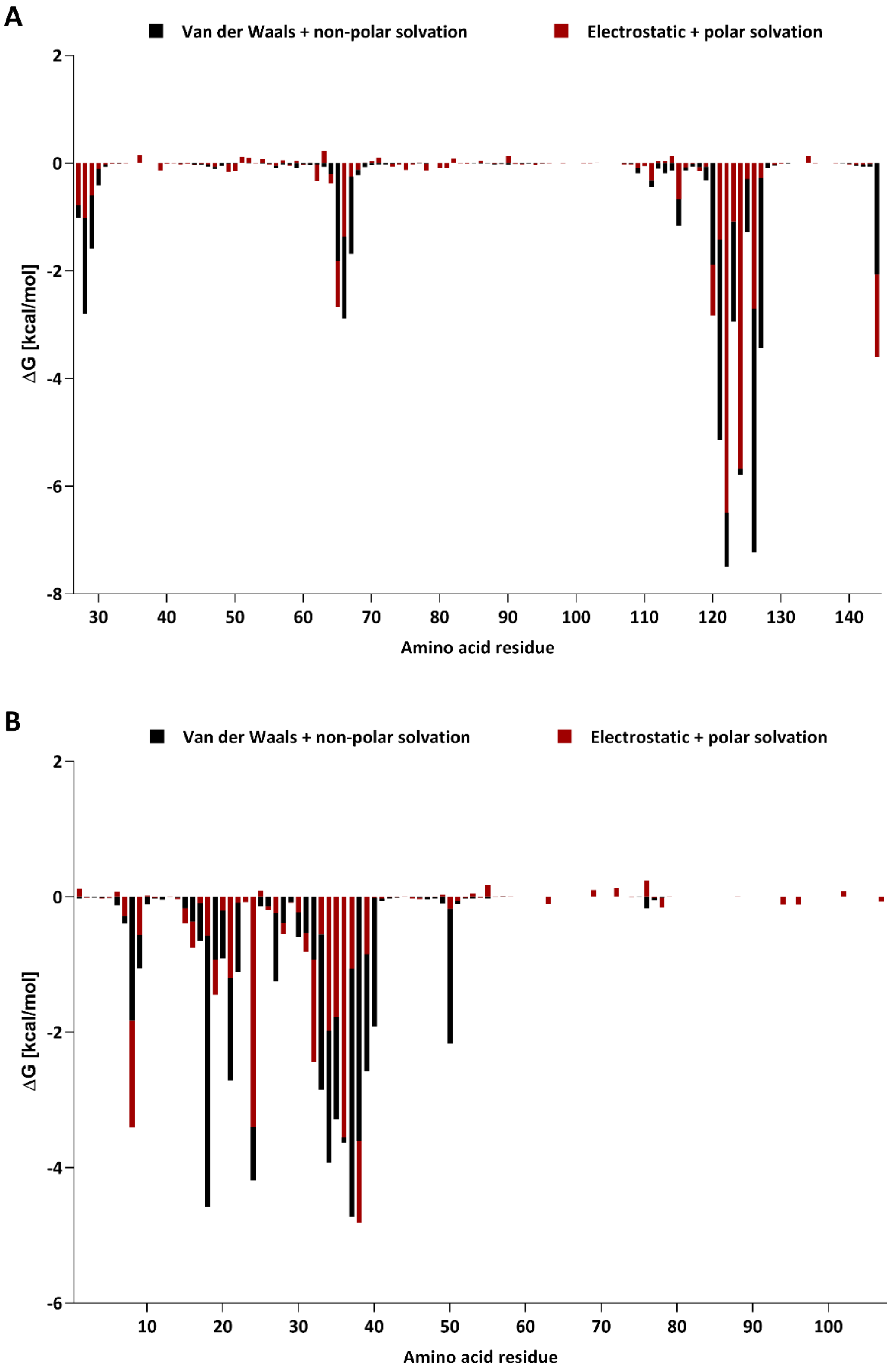

Figure S4. *Pairwise per-residue energy decomposition divided into van der Waals + non-polar and electrostatic + polar contributions for A) CD160 and B) HVEM amino acid residues. The energy values were superimposed for each residue.*

Comparison of the binding affinity obtained from SpS and MM-GBSA

Table S3. *Experimental and theoretical binding affinities for the BTLA/HVEM and HVEM/peptide complexes. Experimental K_d values obtained from SpS were used to calculate the corresponding ΔG, while theoretical ΔG values were obtained from MM-GBSA analysis.*

**protein/protein or protein/peptide complex**	**K_d [µmol]**	**SD**	**ΔG calculated from K_d [kcal/mol]**	**SD**	**ΔG obtained from MM-GBSA [kcal/mol]**	**SD**
BTLA/HVEM	0.31	0.03	-8.89	0.05	-42.49 (from crystal structure)	14.19
HVEM/A5	1.38	0.13	-8.00	0.06	-51.30 (from crystal structure)<br>-22.22 (from NMR)	10.38<br>17.96
HVEM/A4	231	44	-4.96	0.11	-36.37 (from crystal structure)	4.00

The comparison of binding free energies derived from experimental K_d values and MM-GBSA calculations reveals consistent trends across all studied complexes. A5 peptide binds to HVEM significantly stronger than the A4 peptide, as reflected by both the lower K_d and more favourable ΔG values. This observation is supported by MM-GBSA calculations, which also predict a substantially stronger interaction for HVEM/A5 compared to HVEM/A4. Two ΔG values were obtained for the HVEM/A5 complex depending on the initial structure used in the simulations. The model based on the crystal structure yielded a more favourable binding energy (-51.30 kcal/mol) than the model derived from the NMR structure of the peptide (-22.22 kcal/mol). This difference suggests that the A5 peptide, which is flexible in solution, must undergo significant conformational rearrangement upon binding to HVEM. Such structural adaptation likely incurs an energetic penalty, resulting in a less favourable overall ΔG when starting from the NMR-derived structure.

Cell viability assays

The cytotoxicity of the peptides against two cell lines, Jurkat E6.1 and TCS, was examined. To estimate the number of viable cells, the CellTiter-Glo® luminescence-based assay was used, which quantifies adenosine triphosphate (ATP) levels as an indicator of metabolically active cells, directly correlating with cell viability. The assay mechanism relies on the luciferase-luciferin reaction and requires the presence of ATP (produced by living cells), Mg^{2+}

(from the growth medium) and O_2. The process results in energy emission, with a peak intensity at 560 nm (yellow-green light).

The experiments were performed on white 96-well NUNC™ MicroWell™ Nunclon Delta-treated plates (Thermo Fisher Scientific, Waltham, MA, USA) for cell culture. 40 µL of cell suspension were added into each well, ensuring approximately 10,000 (TCS) and 20,000 (Jurkat E6.1) cells per well, and 40 µL of peptide solutions. Peptide samples were dissolved in RPMI 1640 medium supplemented with 10% FBS, penicillin (100 U/mL) and streptomycin (100 µg/mL) (Sigma Aldrich, St. Louis, MO, USA) and final concentrations of peptide in wells was 150, 50, 16.67, 5.56, and 1.85 µM. The wells containing 40 µL of medium and 40 µL of cells (without the peptides) served as control. The plates were incubated for 24 hours at 37°C under a constant supply of CO_2. After the incubation period, 80 µL of CellTiter-Glo® Luminescent Cell Viability Assay solution (Promega Corporation, Madison, WI, USA) was added to each well and incubated for 15 minutes at room temperature. Finally, luminescence was measured using a CLARIOstar Plus plate reader (BMG LABTECH, Ortenberg, Germany). The experiment was performed three times, in triplicate, and the results were analysed with the GraphPad Prism 8 software. Only the three highest peptide concentrations are presented in the figures, as lower concentrations had no measurable effect on cell viability and were therefore omitted.

A)

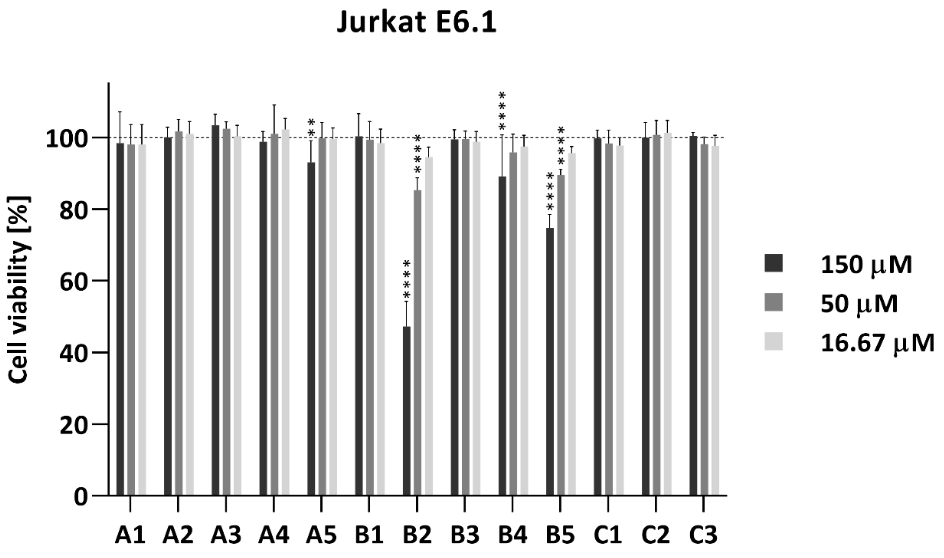

B)

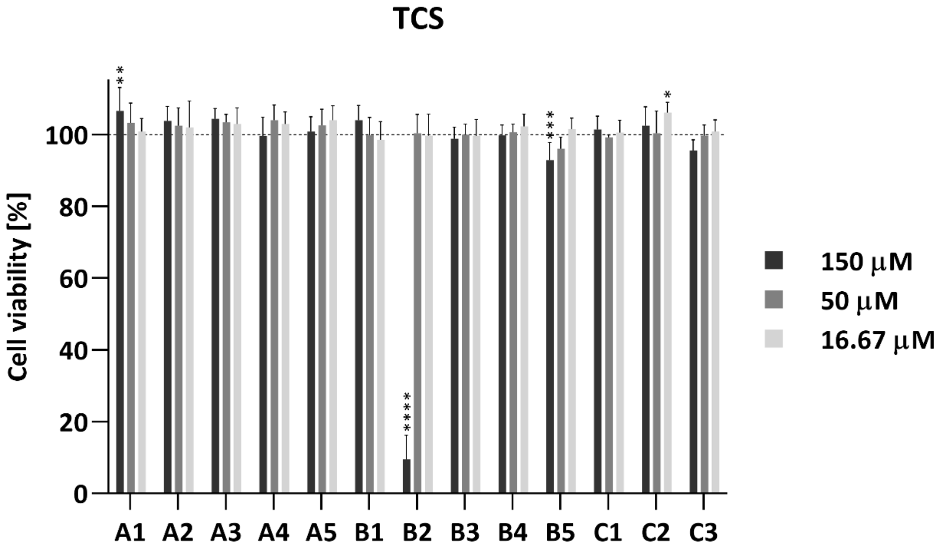

Figure S5. *The influence of the CD160-derived peptides on the viability of A) Jurkat E6.1 and B) TCS cell lines after incubation for 24 hours. Results are shown for three experiments performed independently in triplicate. Data are depicted as mean with SD. Statistical analysis was performed using one-way ANOVA followed by the Dunnet's post-hoc test. ****: $p < 0.0001$, ***: $p < 0.001$, **: $p < 0.01$, *: $p < 0.05$.*

Stability of peptides in cell supernatant

The cell supernatant was obtained by incubating Jurkat E6.1 cell line in RPMI 1640 medium for 24 hours. Following incubation, the cells were centrifuged at 300 × g (1,400 rpm) for 3 minutes at room temperature, and the resulting supernatant was used for the assays.

Peptides dissolved in water at a concentration of 1.5 mg/mL were mixed with cell supernatant and incubated at 37°C with continuous shaking. Samples for analysis were collected at time points 0 and 24 hours. As a control, the peptide was mixed with water rather than cell supernatant. Each sample was precipitated using 1 mL of absolute ethanol, incubated on ice for 15 minutes, and centrifuged at 30,065 × g (18,000 rpm) for 20 minutes at 4°C. The resulting supernatants were transferred to new tubes and evaporated using a vacuum centrifugal concentrator. The samples were then reconstituted in 100 µL of solution A (0.1% TFA in water) and 20 µL of solution B (80% acetonitrile in water with 0.08% TFA), and analysed using RP-HPLC with ELSD-LT detector (Shimadzu, Kyoto, Japan) on a Kromasil C8 analytical column (250 mm × 4.6 mm, 5 µm; Phenomenex, Torrance, CA, USA). A linear gradient from 5% to 100% of solution B in solution A was applied over 60 minutes. Peptide stability was assessed by comparing the peak areas at each time point with the peak area of the control sample at time 0.

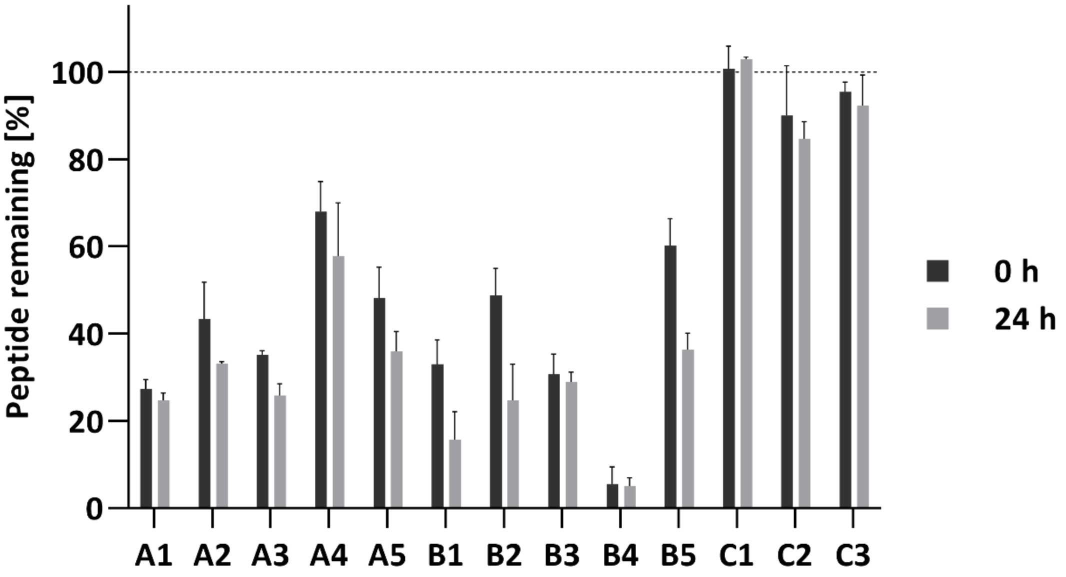

Figure S6. Stability of peptides in Jurkat E6.1 cell supernatant after 0 and 24 hours of incubation, compared to control sample (peptides dissolved in H_2O at time 0). Data are presented as mean with SD from two independent experiments.

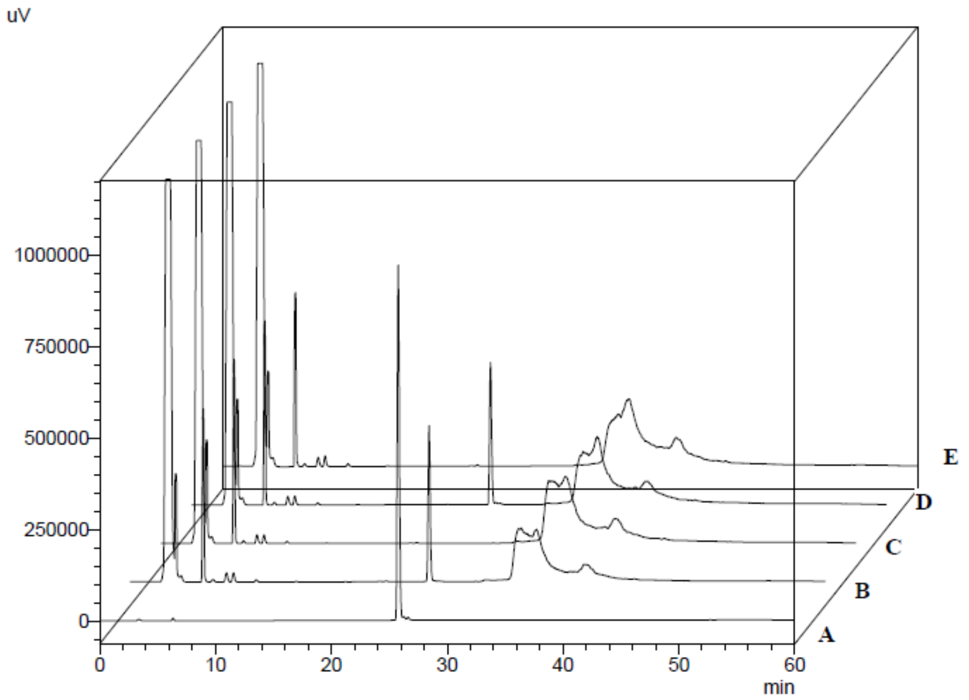

Figure S7. *Comparison of chromatograms obtained for: A) peptide A5 dissolved in H_2O at time 0; B) peptide A5 dissolved in cell culture supernatant at time 0; C) cell culture supernatant at time 0; D) peptide A5 in cell culture supernatant after 24 hours of incubation; E) cell culture supernatant after 24 hours of incubation.*

CD measurement

Far-UV circular dichroism spectra of peptide A5 were measured, in 1 mm pathlength cell on J-815 spectropolarimeter (JASCO Inc., Easton, MD, USA) at 25ºC. The peptide concentration was 150 µg/mL. The scans were performed in the range 195–260 nm. The measurements were performed in duplicate and then averaged.

The CD analyses were initially performed in H_2O and in a commercially available PBS buffer containing 10 mM phosphate buffer, 140 mM sodium chloride, and 2.7 mM potassium chloride, pH 7.4. In H_2O, the peptide A5 exhibited a disordered conformation, as indicated by a minimum around 197 nm, typical for an unstructured peptide. In contrast, in PBS the peptide adopted a β-sheet structure, characterized by a negative band near 214 nm and a positive band around 198 nm (Figure S8A).

The structural ordering observed for the peptide A5 in PBS was initially analysed to assess whether it results from ionic strength effects or from specific interactions with phosphate ions. To gain further insight into the conformational changes of the peptide under different solution conditions, CD spectra were recorded in: H_2O; H_2O containing 2.7 mM KCl, 10 mM

NaCl, 100 mM NaCl, or 140 mM NaCl; 10 mM PBS (phosphate buffer with 140 mM NaCl and 2.7 mM KCl, pH 7.4); and 10 mM sodium phosphate buffer without salts (NaH_2PO_4/Na_2HPO_4, pH 7.4). Based on the obtained results, it was concluded that the salts do not affect the CD spectrum of the A5 peptide. In both PBS containing salts and phosphate buffer without salts, the A5 peptide adopts a β-sheet structure, whereas in water containing KCl or NaCl, the structure remains disordered. These observations indicate that phosphate ions play a key role in promoting the formation of the β-sheet structure in the A5 peptide (Figure S8B).

In the next step, the effect of phosphate buffer saline concentration on the secondary structure of the A5 peptide was analysed. CD spectra were recorded in H_2O and in PBS solutions with increasing phosphate concentrations (0.1, 1, 5, and 10 mM). The spectra revealed that the peptide structure became progressively more ordered with increasing phosphate concentration (Figure S8C). At phosphate concentrations of 5 and 10 mM, the A5 peptide adopted a β-sheet conformation, indicating that this structure is formed preferentially at higher phosphate concentrations.

Finally, the effect of pH on the A5 peptide structure was examined. CD spectra were recorded in 10 mM PBS at pH 6.4, 7.4, and 8.0. The obtained spectra indicated that, within this pH range, the peptide retained its β-sheet structure, demonstrating that the secondary structure is not affected by moderate pH variations (Figure S8D).

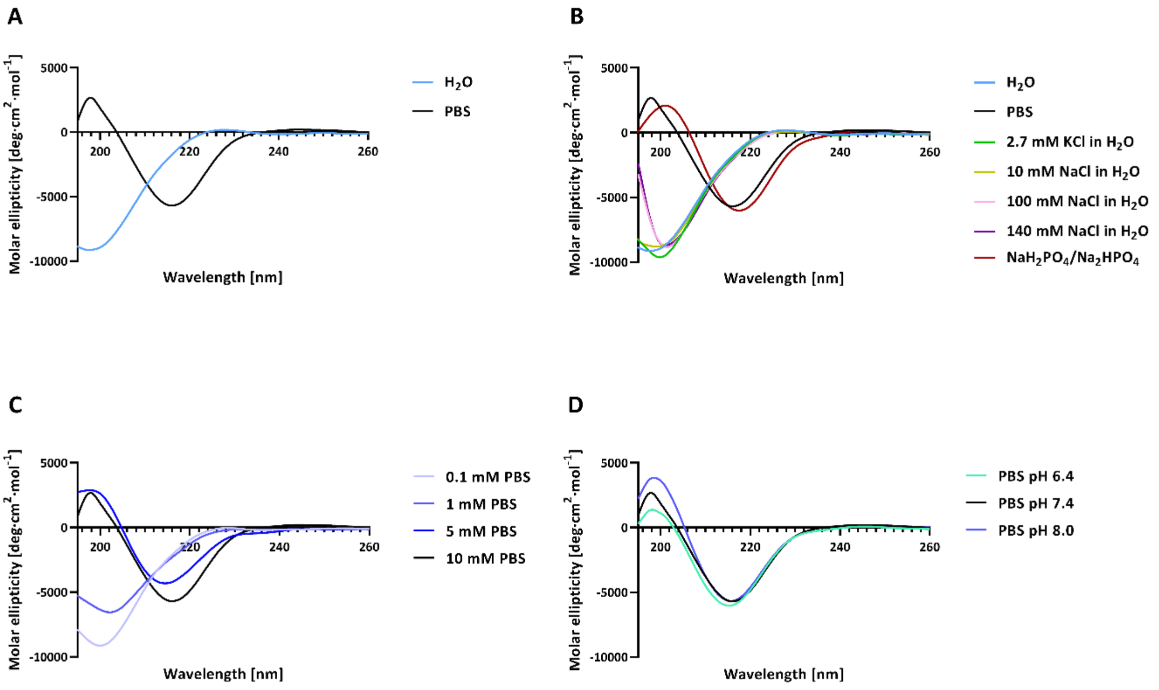

Figure S8. *CD spectra of the A5 peptide under various conditions: A) in H_2O and PBS, and depending on: B) ionic strength, C) PBS concentration, and D) PBS pH.*

NMR measurement

Proton resonances of A5 peptide were assigned using standard procedures based on 2D TOCSY and NOESY datasets (Table S4). The homonuclear NMR spectra were complemented by a heteronuclear 2D ^{1}H–^{13}C HSQC experiment. The C_β chemical shifts of both cysteine residues (~42 ppm) confirmed their oxidized state and the formation of a disulfide bond. The completeness of proton chemical shift assignments reached 97.1%. Sequence-specific assignments were performed in a standard manner, utilizing the Hα–HN and HN–HN regions from 2D ^{1}H-^{1}H NOESY spectra (Figure S9). The narrow chemical shift dispersion of amide protons ($\Delta\delta_{HN}$ = 0.7 ppm), combined with the absence of medium- and long-range interproton distance constraints and the values of $^3J_{N\alpha}$ coupling constants (Figure S10) rules out the presence of a well-defined secondary structure. However, HN–HN (i, i+1) connectivities in the 10–16 fragment of the peptide (residues S120–C126 in CD160), along with chemical shift analysis suggest the presence of turn-like motifs.

The upper interproton distances derived from NOE intensities (116 ^{1}H–^{1}H distance constraints) were incorporated into molecular dynamics simulations using a simulated annealing protocol to model the three-dimensional structure of the peptide. From a total of 1000

generated structures for A5 peptide, 133 were selected based on a total distance penalty not exceeding 0.5 kcal/mol. These structures were subsequently minimized and grouped into conformational families. To assess structural similarity, the root mean square deviation (RMSD) of the backbone atoms within the cyclic region (C3–C16) was used as a distance metric. This approach identified 10 distinct conformational families (Table S5), with one predominant cluster encompassing 67% of the structures. The RMSD of the backbone atoms in the cyclic region of the conformations from the dominant family is 2.329 ± 0.329 Å relative to the average conformation, indicating considerable structural variability despite the clustering algorithm. The Ramachandran plot highlights the dominant conformations within the ensemble of unfolded states. The central region of the peptide may adopt turn or bend structures, aligning well with the NMR data. The average radius of gyration (Rg) for the structures within the dominant family is 9.3 ± 0.5 Å. Analysis of the molecular shape, based on normalized principal moments of inertia (Figure S11), reveals a distribution rather than a single, well-defined conformation, in which the peptide molecule transitions from an elongated to a spherical shape. This change is accompanied by a decrease in Rg value (Figure S11).

Table S4. *Proton chemical shifts for A5 peptide at 298 K.*

Residue	Proton chemical shifts /ppm			
	HN	Hα	Hβ	Others*
Y^1	8.21	4.52	2.88, 2.84	$\underline{CH_3}$CO 1.95, Hδ 7.03, Hε 6.79
Q^2	8.41	4.34	1.91, 2.03	Hγ 2.25, εNH_2 6.87, 7.50
C^3	8.42	4.74	2.99, 3.09	
X^4	8.32	4.25	1.74, 1.84	Hγ 2.06, Hδ 0.93
A^5	8.28	4.33	-	β-CH_3 1.36
R^6	8.30	4.36	1.75, 1.86	Hδ 3.18, Hγ 1.62, ε-NH 7.17
S^7	8.28	4.46	3.86, 3.91	
Q^8	8.38	4.38	2.02, 2.36	Hγ 2.36, εNH_2 6.86, 7.48
K^9	8.38	4.28	1.78, 1.87	Hγ 1.43, 1.47, Hδ 1.69, Hε 3.00, ζ-NH_2 7.53
S^{10}	8.15	4.42	3.88, 3.93	
G^{11}	8.33	3.90, 3.89		
I^{12}	7.87	4.20	1.84	Hγ 1.15, 1.43, δ-CH_3 0.85, γ-CH_3 0.88
R^{13}	8.44	4.36	1.75, 1.81	Hδ 3.18, Hγ 1.57, 1.64, ε-NH 7.17
L^{14}	8.25	4.38	1.55, 1.60	Hγ nd, δ-CH_3 0.84, 0.88
Q^{15}	8.48	4.37	1.95, 2.05	Hγ 2.32, εNH_2 6.80, 7.42
C^{16}	8.40	4.76	2.92	
H^{17}	8.57	4.64	3.03, 3.09	Hδ2 6.96, Hε1 8.51
F^{18}	8.30	4.55	2.87, 2.97	Hδ 7.11, Hε 7.25, Hζ nd
F^{19}	8.23	4.53	2.91, 3.11	Hδ 7.24, Hε 7.31, Hζ nd, CO-$\underline{NH_2}$ 6.79, 7.29

* The amide protons of the side chains of glutamine residues were found, but due to the lack of correlation with the other side chain protons they could not be assigned to specific Gln residues.

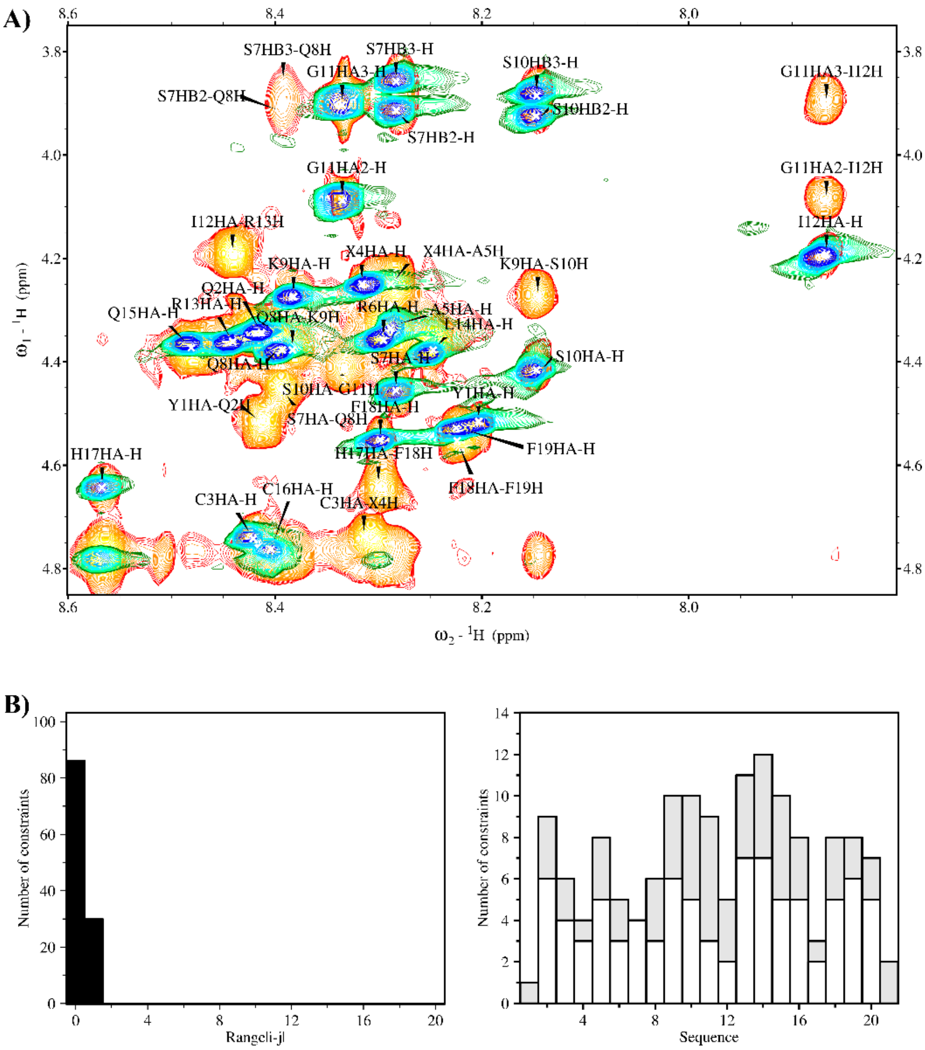

Figure S9. *A) Overlay the HN-Hα region of the TOCSY (green-blue) and NOESY (red-yellow) spectra recorded for A5 at 298 K; B) Distribution of distance restraints. The left plot illustrates the number of distance restraints as a function of the residue index. The right plot displays, for each residue, the number of intra-residual (white) and short-range (grey) restraints.*

A)

B)

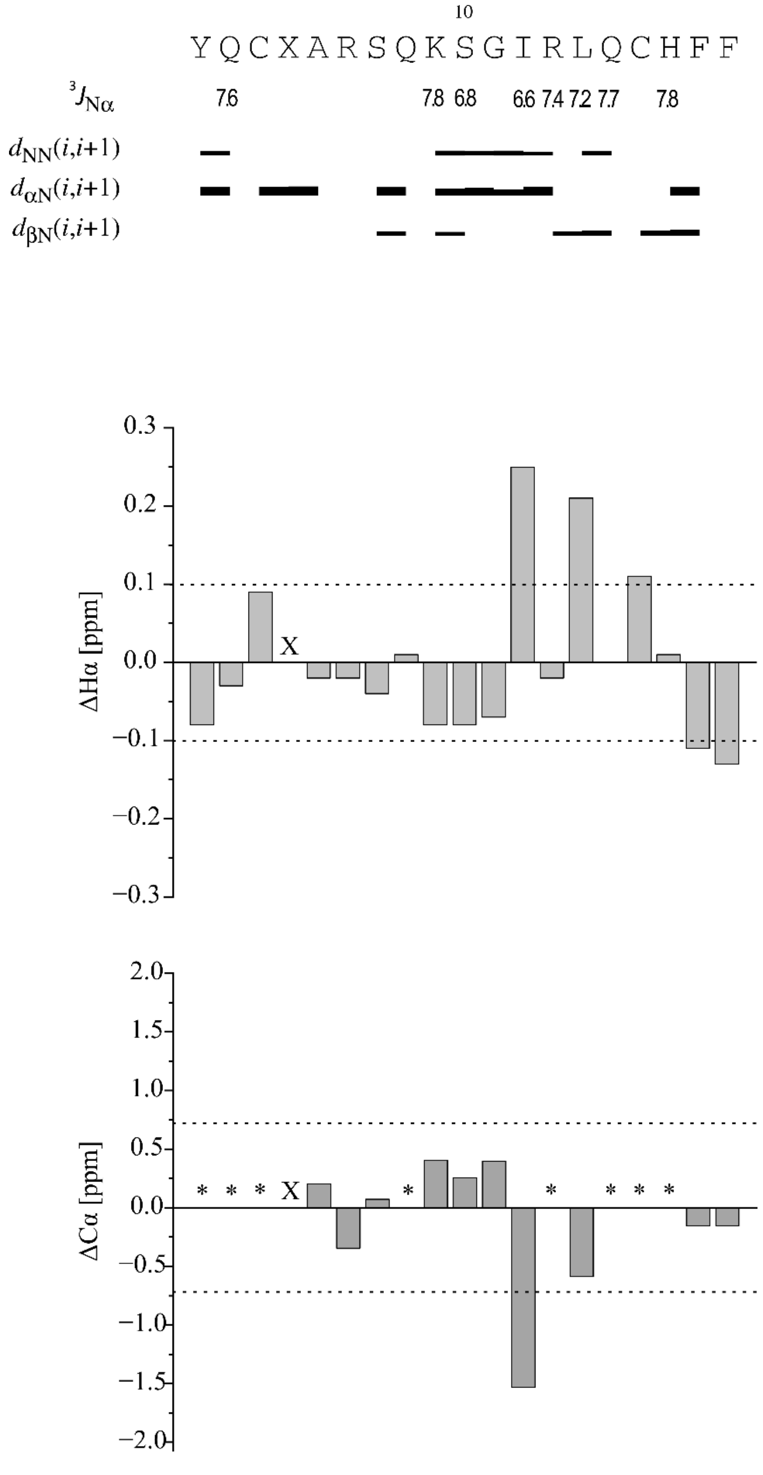

Figure S10. *A) Sequence plots of NOESY distance constraints for A5; B) Hα and Cα chemical shift analysis. Dashed lines represent standard deviation of the referenced chemical shifts. The chemical shift analysis of the 2-aminobutanoic acid residue was not performed due to the absence a chemical shift pattern in the random-coil structure. The Cα chemical shifts marked with an asterisk were not determined due to overlap with the HDO signal.*

Table S5. *Summary of conformational clusters found by using of a hierarchical agglomerative algorithm. AvgDist and Stdev correspond to distance between points in the clusters and standard deviation of points in the cluster, respectively.*

Cluster No.	Frames	Fraction	AvgDist [Å]	Stdev [Å]
1	89	0.669	3.263	0.559
2	16	0.12	3.049	0.498
3	9	0.068	2.999	0.377
4	4	0.03	2.661	0.508
5	3	0.023	2.796	0.662
6	3	0.023	2.743	0.289
7	3	0.023	2.703	0.379
8	3	0.023	2.896	0.316
9	2	0.015	2.643	0
10	1	0.008	0	0

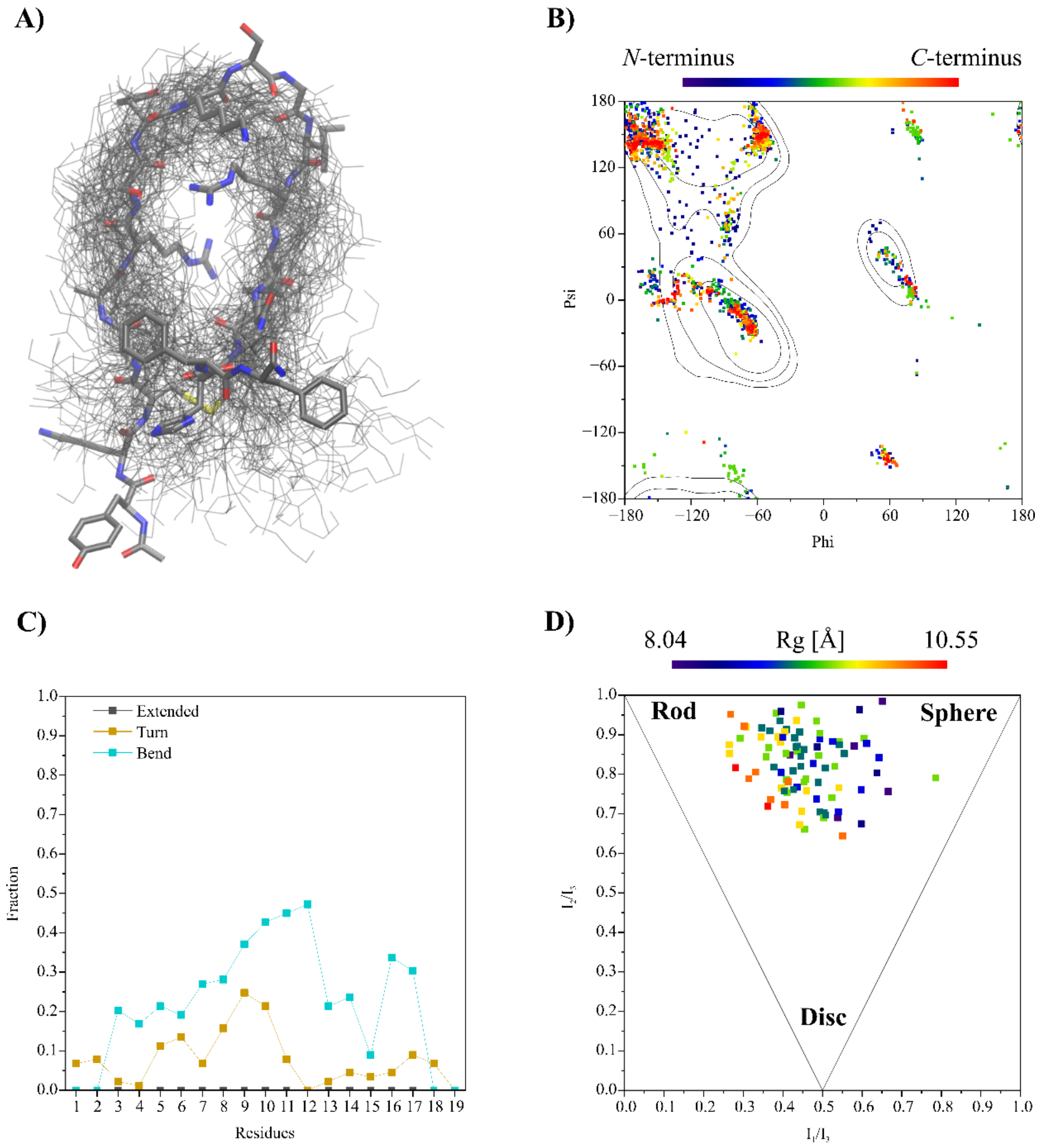

Figure S11. *A) Superimposed conformations; B) Ramachandran plot; C) secondary structure analysis; only extended, turn, and bend structures were observed; other types were absent or negligible; and D) principal moment of inertia (PMI) plot illustrating the three-dimensional shape diversity of the conformations of peptide A5 from a dominant conformational family. RMSD for the backbone atoms from residues C^3 to C^{16} was 2.329±0.329 Å relative to the average conformation. Rg represents the radius of gyration calculated for all heavy atoms.*

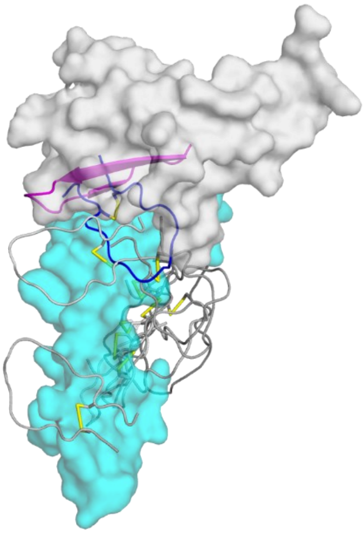

Figure S12. *Structural representation of the BTLA (grey surface)/HVEM (cyan surface) complex (PDB code: 2AW2) with the ten top-ranked peptide A5 binding modes predicted by HDOCK. The model of peptide A5 bound at the same site as BTLA is shown as a dark blue ribbon, while the other models, binding to CRD2 or CRD3 of HVEM, are displayed in different shades of grey. The disulfide bond is represented as yellow sticks. The CD160 fragment (residues 110–128) from the CD160/HVEM complex (PDB code: 6NG3) is shown as a magenta cartoon.*

Table S6. *Percentage of secondary structures calculated using the cpptraj algorithm for the A5 peptide based on molecular dynamics.*

	**α-Helix [%]**	**β-Sheet [%]**	**Random Coil [%]**
**A5 (from crystal structure)**	11.17	17.81	71.02
**A5 (from NMR)**	17.33	0.37	82.31
**A5 (from crystal structure) in complex with HVEM**	15.79	40.28	43.93
**A5 (from NMR) in complex with HVEM**	0.00	16.84	83.16

The structure of peptide A5, obtained from molecular dynamics simulations performed on models from the crystal structure and NMR data, indicates that the crystal-derived peptide exhibits a higher content of β-sheets and a lower degree of disorder compared to the NMR-derived peptide, in which random coil conformations dominate. Upon binding to HVEM, peptide A5 undergoes pronounced structural ordering — in the crystal model, the content of β-

sheets increases substantially and the amount of random coil decreases, whereas in solution simulations of the NMR-derived peptide, only a moderate increase in β-sheet content is observed while the random coil remains predominant. These results suggest that the crystal-derived A5 binds to HVEM adopting a more ordered conformation than the NMR-derived structure, confirming our previous observations regarding the peptide's conformational flexibility.

Table S7. *Entropic contributions (TΔS) calculated from normal mode analysis (NMA) for HVEM in complexes with peptide A5 (obtained from CD160/HVEM crystal stucture and NMR).*

Complex	(TΔS) [kcal/mol]	SD
HVEM/A5 (from NMR)	-28.71	-7.19
HVEM/A5 (from crystal)	-32.27	-1.20

HVEM/A5 complexes (from NMR and crystal structure) exhibited comparable entropy losses upon binding. The entropy loss was slightly smaller for the complex in which the A5 peptide structure was derived from NMR data (-28.71 kcal/mol) compared to the model based solely on the crystal structure (-32.27 kcal/mol). This difference suggests that the A5 peptide may interact with HVEM through a more flexible binding mode in solution, consistent with the conformational variability observed in the NMR data.

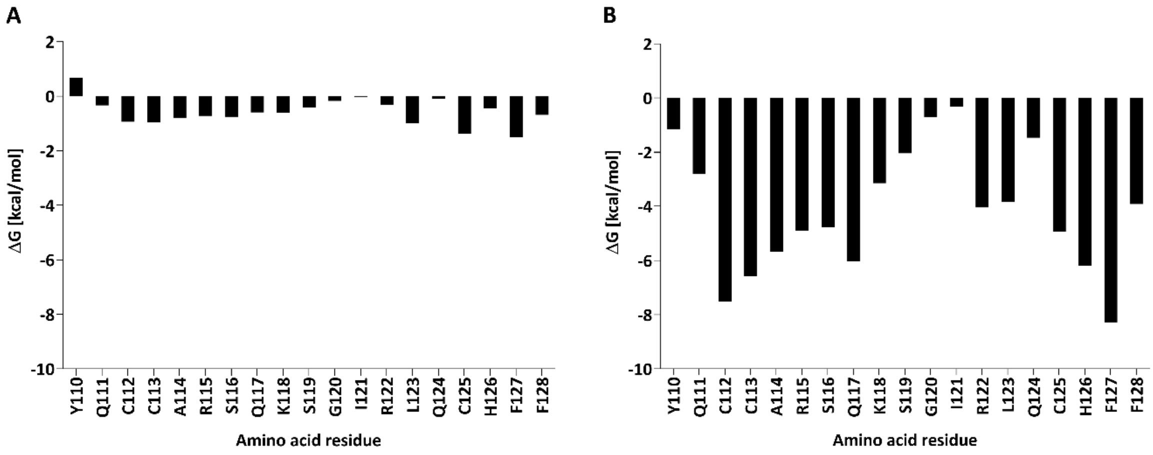

Figure S13. *A) Per-residue and B) pairwise per-residue energy decomposition analysis for peptide A5. The criterion for "strong" interaction energy was defined as an energy of -1 kcal/mol or lower for per-residue decomposition, and -3 kcal/mol or lower for pairwise per-residue decomposition.*

Tabel S8. *Comparison between different methods of free energy change difference of alanine mutation (ΔΔG) obtained with FoldX, with the from MMGBSA alanine scan and from Prodigy.*

**Residue**	**ΔΔG (from FoldX)**	**ΔΔG (from MM-GBSA)**	**ΔΔG (from Prodigy)**
Y110A	-1.03	-0.05	-0.2
Q111A	-0.13	0.36	1.1
C112A	4.12	1.39	0.5
C113A	-0.87	0.46	1.2
A114A	0	-	0
R115A	-0.16	1.19	0.9
S116A	-0.18	0.76	1.1
Q117A	0.73	0.70	1.6
K118A	0.73	0.33	1.4
S119A	-0.53	-0.01	1.1
G120A	-0.04	-	0.9
I121A	0.90	0.02	0.9
R122A	0.18	0.71	1.3
L123A	2.17	0.50	0.9
Q124A	0.27	-0.07	1.2
C125A	3.49	1.48	0.7
H126A	-2.67	0.29	1.2
F127A	0.60	1.42	1.5
F128A	-0.18	-	0.9

The results obtained from FoldX and MM-GBSA analyses indicate that cysteine residues, which form disulfide bonds, are critical for the stability of the A5 peptide. These residues maintain the structural integrity of the peptide, and their substitution with alanine leads to peptide unfolding, as reflected by the positive ΔΔG values. In contrast, PRODIGY does not explicitly account for disulfide bonds, which explains the poor correlation of its ΔΔG values with the other methods. FoldX analysis further suggests that the substitutions Y110A (ΔΔG = -1.03 kcal/mol) and H126A (ΔΔG = -2.67 kcal/mol) could potentially enhance binding to HVEM. However, MM-GBSA analysis does not indicate any alanine substitution that would favorably affect binding, a result that is consistent with PRODIGY predictions.

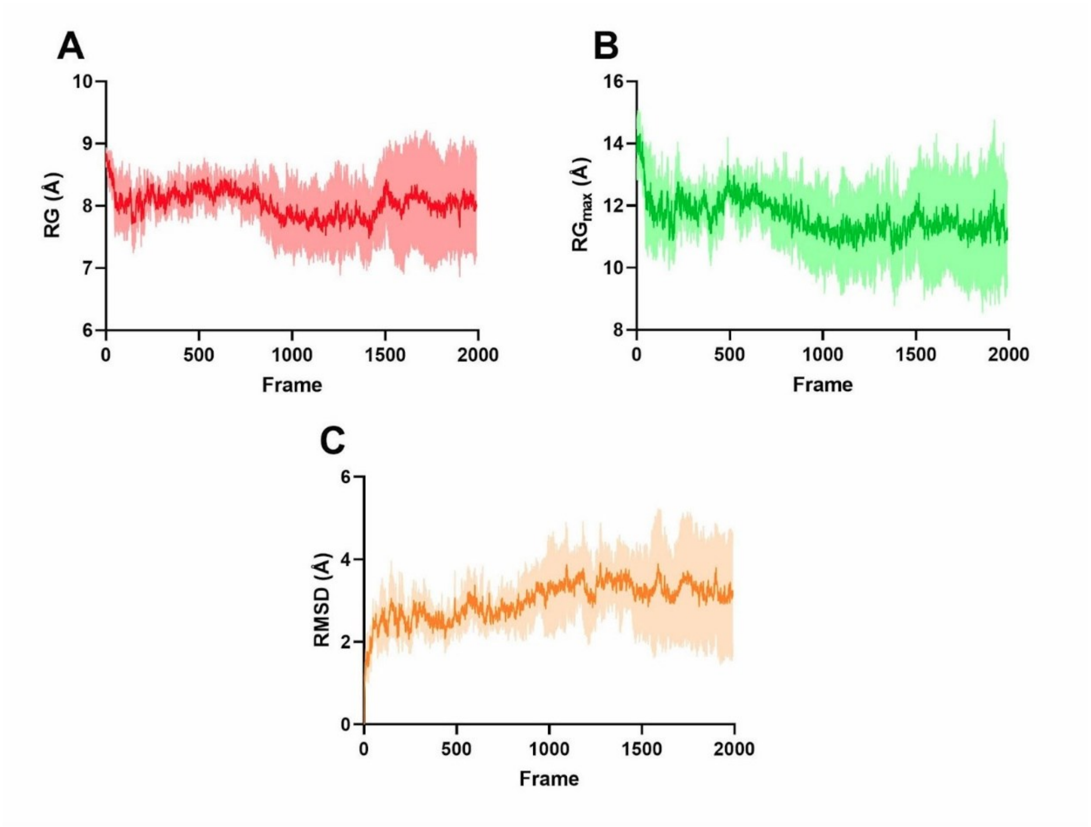

Figure S14. *A) Rg, B) Rgmax, and C) RMSD as a function of frame for the A5 peptide during the simulation.*

For the A5 peptide, the Rg initially decreases and then increases after approximately 1500 frames, after which it remains approximately constant. In the case of Rgmax a decrease in value is observed, and after around the 1000th frame it oscillates around a constant value. The RMSD of the peptide A5 increases steadily until about the 1500th frame, after which it oscillates around a constant value.

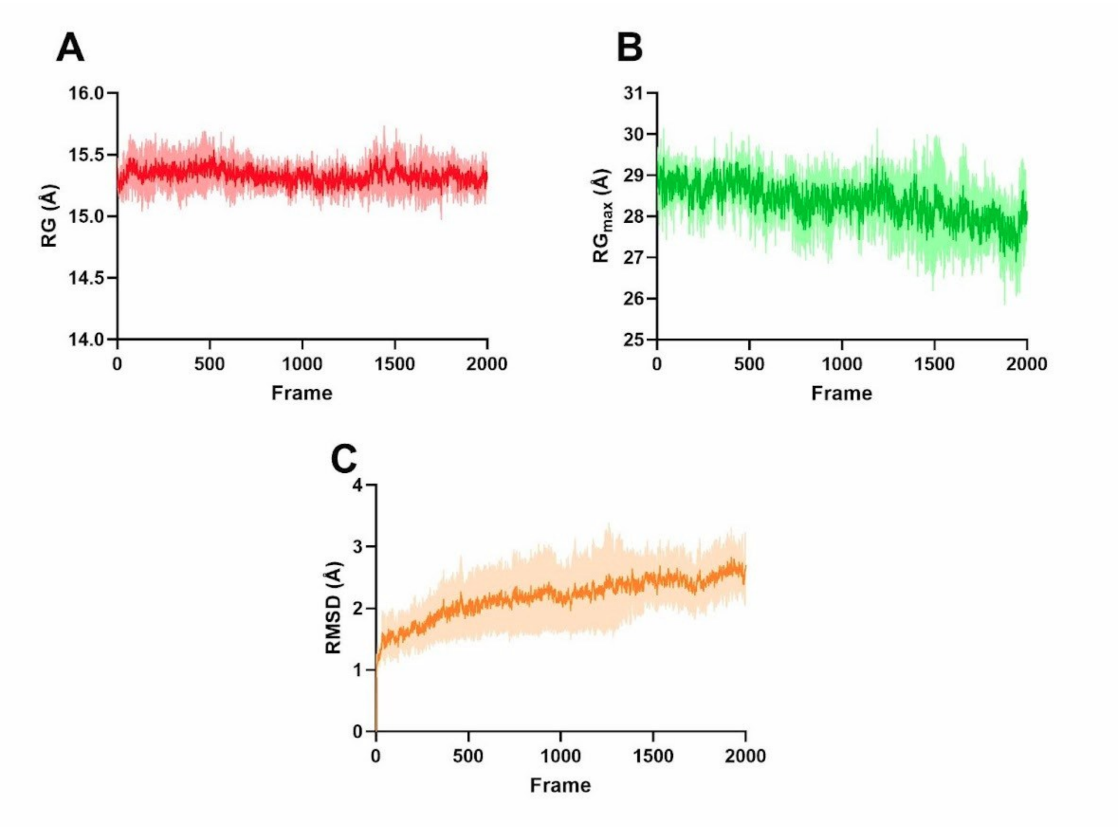

Figure S15. *A) Rg, B) Rgmax, and C) RMSD as a function of frame for the CD160 protein during the simulation.*

For the CD160 protein the Rg remains at constant level throughout the whole simulation. In case of Rgmax, a decrease in value is observed, and after approximately the 1500th frame it oscillates around a constant value. The RMSD of CD160 increases steadily throughout the simulation; however, after the 1000th frame, the increase becomes marginal (less than 0.5 Å).

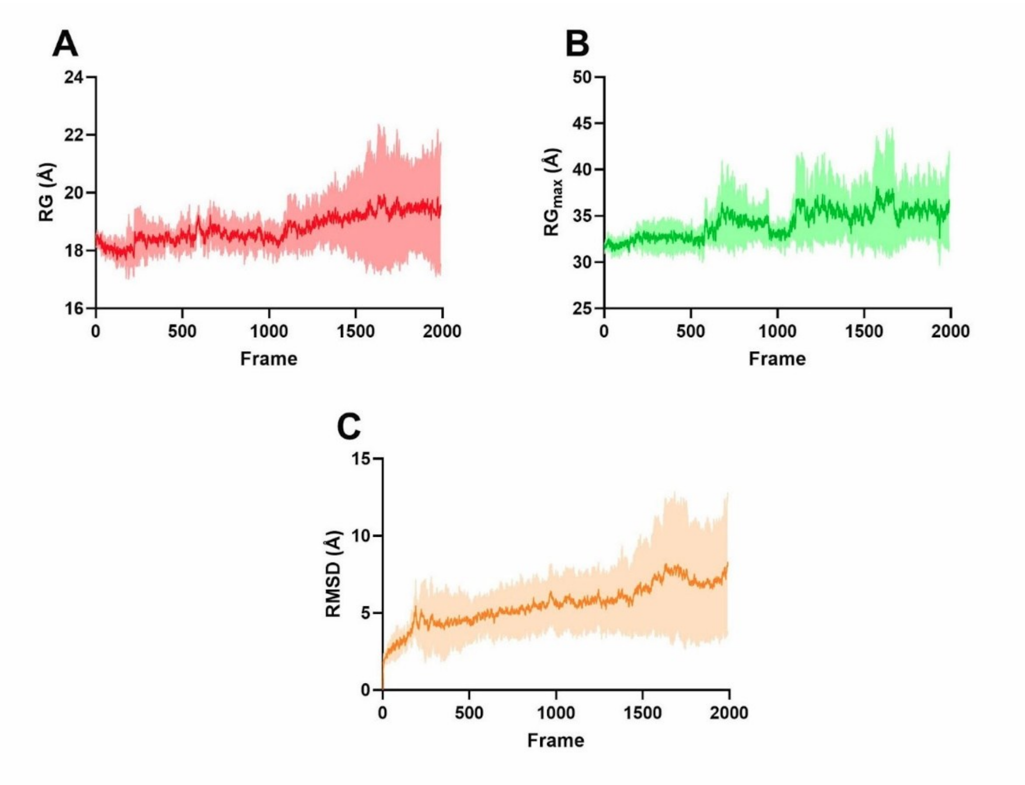

Figure S16. *A) Rg, B) Rgmax, and C) RMSD as a function of frame for the HVEM/A5 complex during the simulation.*

In the case of the HVEM/A5 complex, the Rg reaches convergence after 1500 frames, whereas Rgmax converges just after the 1000th frame. The RMSD of the complex reaches convergence around the 1700th frame, and a decrease in the average RMSD value is observed.

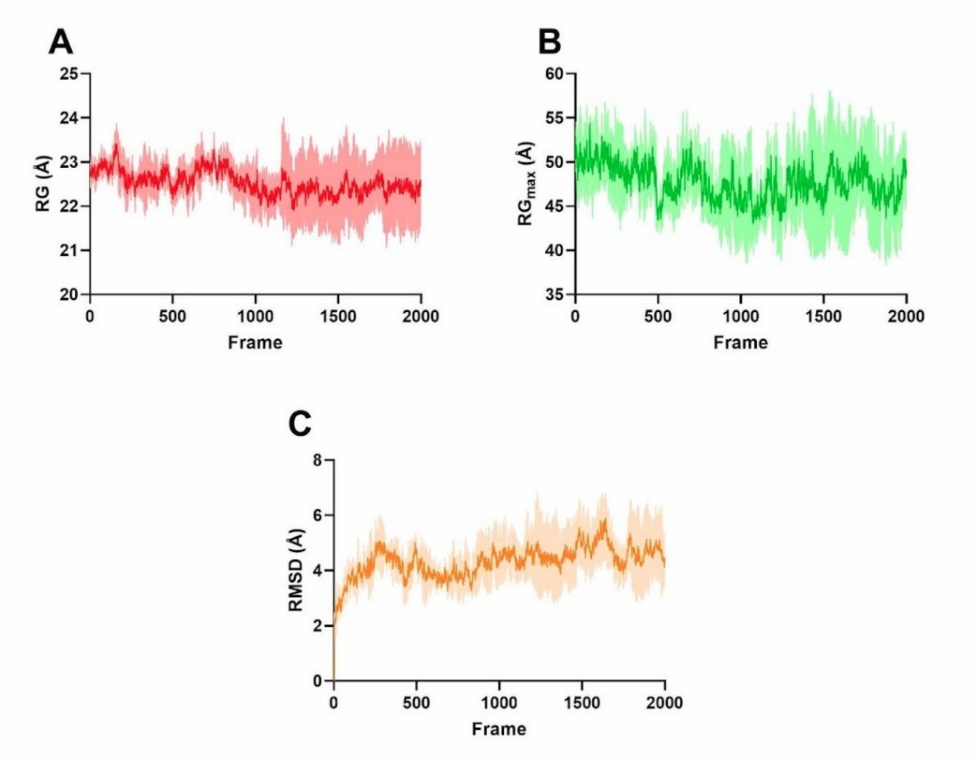

Figure S17. *A) Rg, B) Rgmax, and C) RMSD as a function of frame for the CD160/HVEM complex during the simulation.*

In the case of the CD160/HVEM complex, both Rg and Rgmax converge approximately around the 1000th frame. The RMSD of the complex reaches convergence very quickly, around the 400th frame and only small fluctuations in the RMSD value are observed thereafter.
